# Neurodegenerative disease in *C9orf72* repeat expansion carriers: population risk and effect of *UNC13A*

**DOI:** 10.1093/brain/awaf269

**Published:** 2025-07-19

**Authors:** Jiali Gao, Andrew G L Douglas, Christos V Chalitsios, Jakub Scaber, Kevin Talbot, Martin R Turner, Alexander G Thompson

**Affiliations:** Nuffield Department of Clinical Neurosciences, University of Oxford, Oxford OX3 9DU, UK; Nuffield Department of Clinical Neurosciences, University of Oxford, Oxford OX3 9DU, UK; Oxford Centre for Genomic Medicine, Oxford University Hospitals NHS Foundation Trust, Oxford OX3 9DU, UK; Nuffield Department of Clinical Neurosciences, University of Oxford, Oxford OX3 9DU, UK; Nuffield Department of Clinical Neurosciences, University of Oxford, Oxford OX3 9DU, UK; Nuffield Department of Clinical Neurosciences, University of Oxford, Oxford OX3 9DU, UK; Nuffield Department of Clinical Neurosciences, University of Oxford, Oxford OX3 9DU, UK; Nuffield Department of Clinical Neurosciences, University of Oxford, Oxford OX3 9DU, UK

**Keywords:** amyotrophic lateral sclerosis, frontotemporal dementia, C9orf72, neurodegeneration, UNC13A

## Abstract

The *C9orf72* hexanucleotide repeat expansion (HRE) is the most common monogenetic cause of amyotrophic lateral sclerosis (ALS) and frontotemporal dementia (FTD). Neurodegenerative disease incidence in *C9orf72* HRE carriers has been studied using cohorts from disease-affected families or by extrapolating from population disease incidence, potentially introducing bias.

Age-specific cumulative incidence of ALS and dementia was estimated using Kaplan–Meier and competing risk models in *C9orf72* HRE carriers compared to matched controls in UK Biobank. Risk modification by *UNC13A* genotype was examined.

Of 490 331 individuals with valid genetic data, 701 had >100 repeats in *C9orf72* [median age 55 (interquartile range 48–62), follow-up 13.4 years (12.3–14.1)]. The cumulative incidence of ALS or dementia was 66% (95% confidence interval 57%–73%) by age 80 in *C9orf72* HRE carriers versus 5.8% (4.5%–7.0%) in controls, or 58% (50%–64%) versus 5.1% (4.1%–6.4%), accounting for the competing risk of other-cause mortality. Forty-one per cent of dementia incidence accrued between age 75–80. C-allele homozygosity at rs12608932 in *UNC13A* increased ALS or dementia risk in *C9orf72* HRE carriers [hazard ratio 1.81 (1.18–2.78)].

*C9orf72* HRE disease was incompletely penetrant in this population-based cohort, with risk modified by *UNC13A* genotype. This has implications for counselling at-risk individuals and modelling expected phenoconversion for prevention trials.

## Teaser

The *C9orf72* hexanucleotide repeat expansion is associated with ALS and certain forms of dementia, though its exact penetrance remains unclear. In a UK population-based cohort of 701 people carrying this variant, Gao *et al.* found that 66% developed ALS or dementia by age 80, with implications for genetic counselling.

## Introduction

A hexanucleotide repeat expansion (HRE) in an intronic region of *C9orf72* is the most common genetic cause of amyotrophic lateral sclerosis (ALS) and frontotemporal dementia (FTD) in European-ancestry populations, present in around 10% of ALS and FTD cases.^1-3^ Individuals with *C9orf72* ALS or FTD are indistinguishable from their sporadic counterparts, but, in aggregate, have a younger onset,^2,4^ more equal sex distribution^5^ and shorter survival.^2,4,5^ The effect of repeat length on phenotype remains uncertain.^6^  *C9orf72* HRE has also been reported in parkinsonism,^7,8^ amnestic dementia^9,10^ and Huntington’s disease-like syndrome,^9^ but, given a population prevalence of 1 in 700,^9^ the association between *C9orf72* HRE and these phenotypes is unresolved.

Accurately defining disease incidence in *C9orf72* HRE carriers has implications for genetic counselling, the mechanistic understanding of *C9orf72*-related neurodegeneration and the planning of preventative trials.^11,12^ Estimates have varied widely due to methodological differences. Cohort studies have estimated the penetrance of ALS-FTD in *C9orf72* HRE carriers as near-complete by age 80,^1,13^ but are limited by sample size and biased by recruitment from families with higher penetrance. Indirect estimates calculated from the incidence of ALS in first-degree relatives of *C9orf72* HRE carriers or from the population incidence of ALS-FTD and population prevalence of *C9orf72* HRE have suggested a penetrance of 24%–33%,^14,15^ but are reliant on the accuracy of these underlying statistics and do not include other potential disease phenotypes.

Family history is not universal in *C9orf72*-associated ALS and FTD cases. The marked variability in penetrance between families implies that genetic modifiers, particularly common variants, may influence disease risk.^14^ Among known ALS and FTD risk loci, common polymorphisms in the *UNC13A* gene are notably associated with a modestly increased risk of both diseases,^16^  ^,17^ with convincing mechanistic evidence of pathogenicity in TDP-43 proteinopathies.^18^ In particular, homozygosity for the C allele at rs12608932 has been linked to more aggressive disease and represents a strong candidate modifier of ALS-FTD risk in *C9orf72* HRE carriers.^19^ This study aimed to determine the incidence of ALS, dementia and parkinsonism in *C9orf72* HRE carriers in a prospective population-based cohort study, thereby addressing the limitations of previous study designs. The effect of rs12608932 in *UNC13A* on this risk was also examined.

## Materials and methods

### Participants

The UK Biobank is a prospective, population-based cohort study of 502 625 participants, aged 40–69.^20^ Approximately 9.2 million invitations were sent to individuals living within 40 km from one of 22 assessment centres and 502 625 individuals (5.5% response rate) attended a baseline assessment, consisting of touchscreen questionnaire, interview, physical assessments and biological sampling (blood, urine and saliva). Whole genome sequencing was performed on blood samples from 490 640 participants using Illumina NovaSeq technology, from which *C9orf72* HRE carriers were identified using ExpansionHunter,^21^ with a cut-off of >100 repeats to prioritize specificity (Supplementary material, ‘Methods’ section, ‘ExpansionHunter’). These individuals are referred to as ‘*C9orf72* HRE carriers’ henceforth. As a sensitivity analysis, analyses were repeated, including all individuals with >30 repeats, the commonly used pathogenicity threshold. Controls were restricted to individuals with <30 repeats throughout. Genotyping was also performed using Affymetrix (now part of ThermoFisher Scientific), from which rs12608932 genotype was extracted (Supplementary material, ‘Methods’ section).

Participants also consented to linkage of hospital episode statistics (HES) and death registry data. These data are censored on 31 Oct 2022 in England, 31 Aug 2022 in Scotland and 31 May 2022 in Wales. A small proportion of individuals (1297, 0.26%) were knowingly lost to follow-up prior to this, mostly due to withdrawal of consent or migration overseas.

### Outcomes

Incident ALS, FTD, any-cause dementia (including FTD) and parkinsonism diagnoses were extracted from linked HES and death registry data (Supplementary material, ‘Methods’ section, ‘Neurodegenerative disease outcomes’). Participants with prevalent ALS, dementia or parkinsonism at recruitment were excluded.

### Statistical analysis

*C9orf72* HRE carriers and non-carriers were compared using chi-squared tests for categorical variables and Mann-Whitney U-tests for continuous variables, with Bonferroni adjustment for multiple testing.

Age-specific cumulative incidences of neurodegenerative diseases were estimated using both Kaplan–Meier models, censoring at the end of follow-up or death, and models considering other-cause mortality as a competing risk. *C9orf72* HRE carriers were compared by log-rank testing to controls matched by age, sex, Townsend deprivation index (TDI) and ethnicity (white, non-white; Supplementary material, ‘Methods’ section, ‘Matching’ and Supplementary Table 1). Analyses were also stratified by sex, BMI, smoking, alcohol and highest education level to examine potential effect modification by these factors and subset by data source of diagnosis. The association between expansion size and disease variables was examined using simple linear regression.

The impact of rs12608932 genotype on neurodegenerative disease incidence was investigated using Kaplan–Meier analyses and Cox regression, in the whole cohort and restricted to *C9orf72* HRE carriers. Cox models utilized age as the timescale and were adjusted for sex, age at recruitment and Townsend deprivation index, with robust standard errors calculated using a sandwich estimator clustered by assessment centre. As a sensitivity analysis, models were additionally adjusted for the first 15 genetic principal components and clustered by genetic batch. The interaction between rs12608932 genotype and *C9orf72* HRE status was investigated by inclusion of an interaction term in the whole cohort.

## Results

### Baseline characteristics

Among 490 331 individuals with valid genetic data, 701 had at least one *C9orf72* allele with >100 repeats, indicating a prevalence of 143 per 100 000 [95% confidence interval (CI) 132–154], and two were homozygous for the expansion. Median follow-up of *C9orf72* HRE carriers was 13.4 years [interquartile range (IQR) 12.3–14.1]. Three had prevalent neurodegenerative diagnoses and were excluded.

Compared to unmatched controls with <30 repeats, *C9orf72* HRE carriers without prevalent neurodegenerative diagnoses were younger [median 55 (IQR 48–62) versus 58 (50–63) years, *P* < 0.001], more likely of white ethnicity (97.9% versus 94.2%, *P* < 0.001), never-smokers (61.2% versus 54.4%, *P* = 0.011), non-alcohol-drinkers (10.2% versus 8.0%, *P* = 0.003) and of lower body mass index [BMI; 25.3 (23.0–28.7) versus 26.7 (24.1–29.9) kg/m^2^, *P* < 0.001] (Supplementary Table 2). After matching by age, sex, TDI and ethnicity, differences in smoking, alcohol and BMI remained (Supplementary Table 3). Five individuals with missing matching data were excluded, leaving 693 carriers for further analysis.

### Age-specific incidence of *C9orf72* HRE-associated diseases

**Figure 1:**
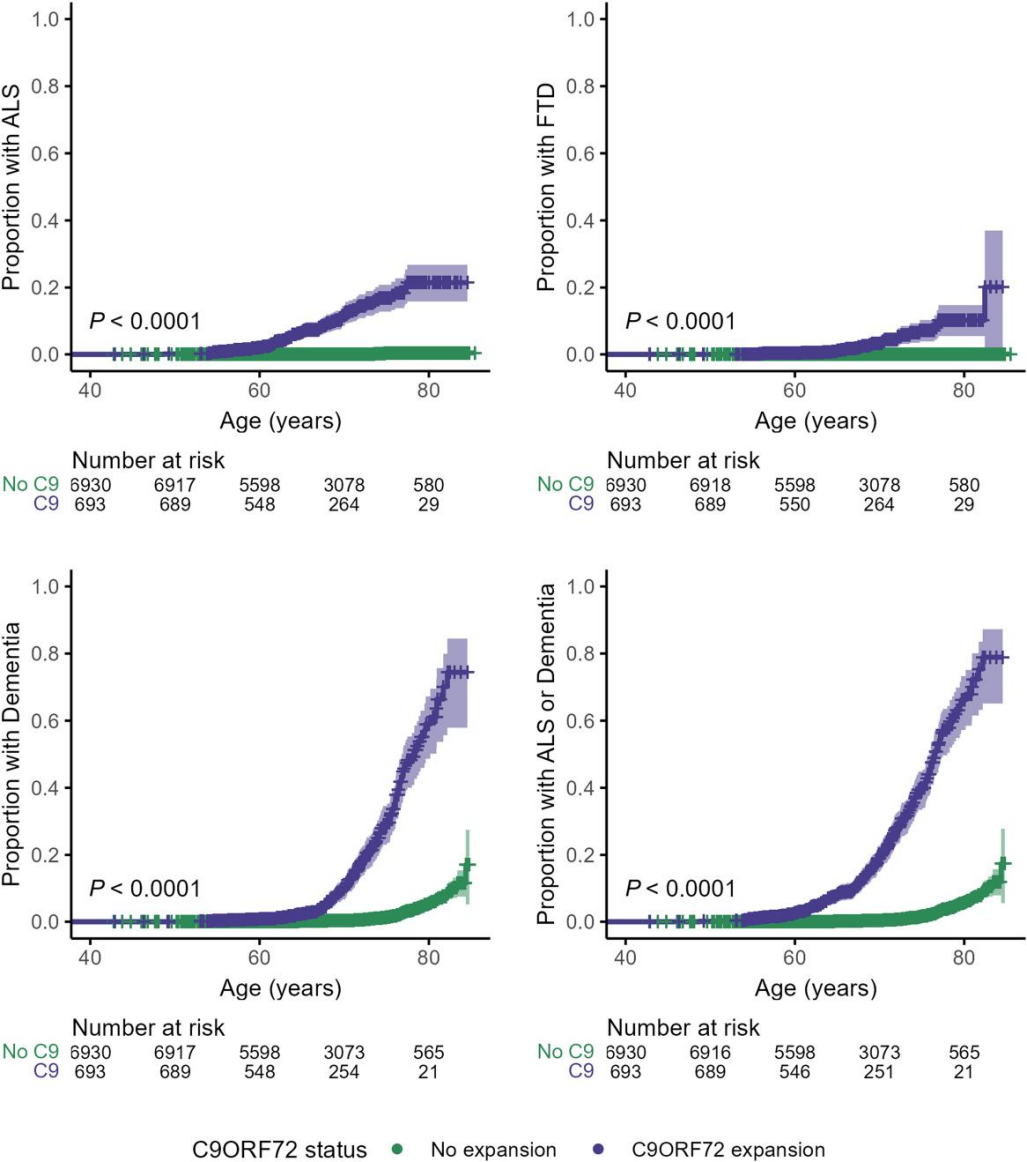
**Age-specific cumulative incidences of ALS and dementias in *C9orf72* HRE carriers compared to matched controls.** Kaplan–Meier plots of cumulative incidences of ALS, FTD, dementia and a combined outcome of ALS and dementia by age in individuals carrying the *C9orf72* HRE (purple, *n* = 693) compared to age, sex, Townsend deprivation index and ethnicity-matched controls (green, *n* = 6930). Shaded bands indicate 95% confidence intervals. *P*-values were calculated by the log-rank test. Tables below represent the number of individuals at risk at a given time point (No C9 = No expansion; C9 = *C9orf72* HRE expansion). Dementia refers to any-cause dementia (including FTD). ALS = amyotrophic lateral sclerosis; FTD = frontotemporal dementia; HRE = hexanucleotide repeat expansion.

Compared to matched controls, *C9orf72* HRE carriers had a higher cumulative incidence by age 80 of ALS [21% (95% CI 16%–27%) versus 0.4% (0.1%–0.7%), *P*_log-rank_ < 0.001], FTD [10% (5.4%–15%) versus < 0.1% (0%–0.2%), *P*_log-rank_ <0.001] and any-cause dementia [59% (49%–67%) versus 5.4% (4.1%–6.7%), *P*_log-rank_ <0.001] (Table 1 and Fig. 1). Adjusting for competing risk of other-cause mortality before disease onset reduced this to 18% (14%–22%) for ALS, 7.3% (4.7%–11%) for FTD and 44% (37%–51%) for any-cause dementia (Supplementary Table 4 and Supplementary Fig. 1). Fifteen *C9orf72* HRE carriers [6.1% (2.3%–9.7%)] had recorded diagnoses of both ALS and dementia by age 80 compared to only one control [<0.1% (0%–<0.1%)]. Of the total dementia incidence linked to *C9orf72*, 41% was accrued between ages 75–80.

**Table 1:** Age-specific cumulative incidences of ALS and dementias in *C9orf72* HRE carriers compared to matched controls

*C9orf72* status	Outcome	Incidence by 65 years% (95% CI)	Incidence by 70 years% (95% CI)	Incidence by 75 years% (95% CI)	Incidence by 80 years% (95% CI)
No C9 exp	ALS	<0.1 (0–<0.1)	<0.1 (0–<0.1)	0.3 (<0.1–0.5)	0.4 (0.1–0.7)
C9 exp	ALS	6.5 (4.3–8.5)	12 (8.4–15)	17 (13–21)	21 (16–27)
No C9 exp	FTD	<0.1 (0–0.1)	<0.1 (0–0.1)	<0.1 (0–0.2)	<0.1 (0–0.2)
C9 exp	FTD	0.9 (0.1–1.8)	3.2 (1.4–5.0)	7.1 (3.9–10)	10 (5.4–15)
No C9 exp	Dementia	0.2 (<0.1–0.3)	0.4 (0.2–0.5)	1.4 (1.0–1.9)	5.4 (4.1–6.7)
C9 exp	Dementia	2.2 (1.0–3.5)	9.8 (6.7–13)	29 (23–35)	59 (49–67)
No C9 exp	ALS or Dementia	0.2 (<0.1–0.3)	0.4 (0.2–0.6)	1.7 (1.2–2.2)	5.8 (4.5–7.0)
C9 exp	ALS or Dementia	7.7 (5.4–9.9)	19 (15–23)	39 (33–45)	66 (57–73)
No C9 exp	ALS and Dementia	<0.1 (0–<0.1)	<0.1 (0–<0.1)	<0.1 (0–<0.1)	<0.1 (0–<0.1)
C9 exp	ALS and Dementia	1.1 (0.2–2.1)	2.4 (0.8–3.9)	3.9 (1.6–6.2)	6.1 (2.3–9.7)
No C9 exp	Parkinsonism	0.1 (<0.1–0.2)	0.2 (<0.1–0.4)	0.9 (0.6–1.3)	2.4 (1.5 -3.2)
C9 exp	Parkinsonism	0.3 (0–0.8)	0.6 (0–1.4)	3.0 (0.6–5.4)	4.3 (0.8–7.7)

Numbers rounded to two significant figures. C9 exp = participants carrying at least one *C9orf72* HRE allele of over 100 repeats (*n* = 693); No C9 exp = participants homozygous for unexpanded *C9orf72* (<30 repeats) matched by age, sex, Townsend deprivation index and ethnicity (*n* = 6930); ALS = amyotrophic lateral sclerosis; CI = confidence interval; FTD = frontotemporal dementia; HRE = hexanucleotide repeat expansion.

Overall, the combined cumulative incidence of ALS and any-cause dementia by age 80 was 66% (57%–73%) in *C9orf72* HRE carriers compared to 5.8% (4.5%–7.0%) in controls, assuming survival to this age, or 58% (50%–64%) in carriers compared to 5.1% (4.1%–6.4%) in controls accounting for other cause mortality.

*C9orf72* HRE carriers showed significantly different parkinsonism incidence compared to controls by log rank testing (*P* = 0.034), but no higher cumulative incidence by age 80 [4.3% (0.8%–7.7%) versus 2.4% (1.5%–3.2%); Supplementary Fig. 2].

Among ALS or dementia patients, *C9orf72* HRE carriers were younger at first recorded diagnosis compared to age and sex-matched non-carriers [ALS: median age 64.9 (95% CI 63.4–67.8) versus 68.7 (66.2–70.8), *n* = 72/group, *P*_log-rank_ = 0.020; dementia: 72.2 (71.3–73.7) versus 74.7 (74.0–75.6), *n* = 120/group, *P*_log-rank_ < 0.001; Supplementary Fig. 3]. Only eight *C9orf72* HRE carriers had a diagnosis of parkinsonism and no significant difference in age of diagnosis was detected (Supplementary Fig. 3).

Stratification by sex, BMI, smoking, alcohol and highest educational qualification demonstrated no interaction between these variables and *C9orf72* status in determining ALS and dementia incidence (Supplementary Fig. 4). Analyses using only hospital diagnoses or only death certificate diagnoses are presented in Supplementary Fig. 5 and Supplementary Table 5.

Among 110 individuals identified to carry between 30 and 100 repeats in *C9orf72*, the cumulative incidence by age 80 of ALS was only marginally higher than in matched controls by Kaplan–Meier modelling [2.8% (0%–6.7%) versus 0.5% (0%–1.5%), *P*_log-rank_ < 0.001; Supplementary Table 6 and Supplementary Fig. 6]. The cumulative incidence of any-cause dementia was 16% (1.2%–29%) compared to 5.4% (2.45%–8.2%) in matched controls (*P*_log-rank_ = 0.004). No cases of FTD were recorded in the 30–100 repeat group.

Cumulative incidence of ALS and dementia in individuals carrying more than 30 repeats in *C9orf72* was 58% (50%–65%) at age 80 compared to 5.8% (4.6%–7.0%) in controls (*P*_log-rank_ < 0.001) (Supplementary Table 7 and Supplementary Fig. 7), assuming survival to this age.

### Expansion size

**Figure 2:**
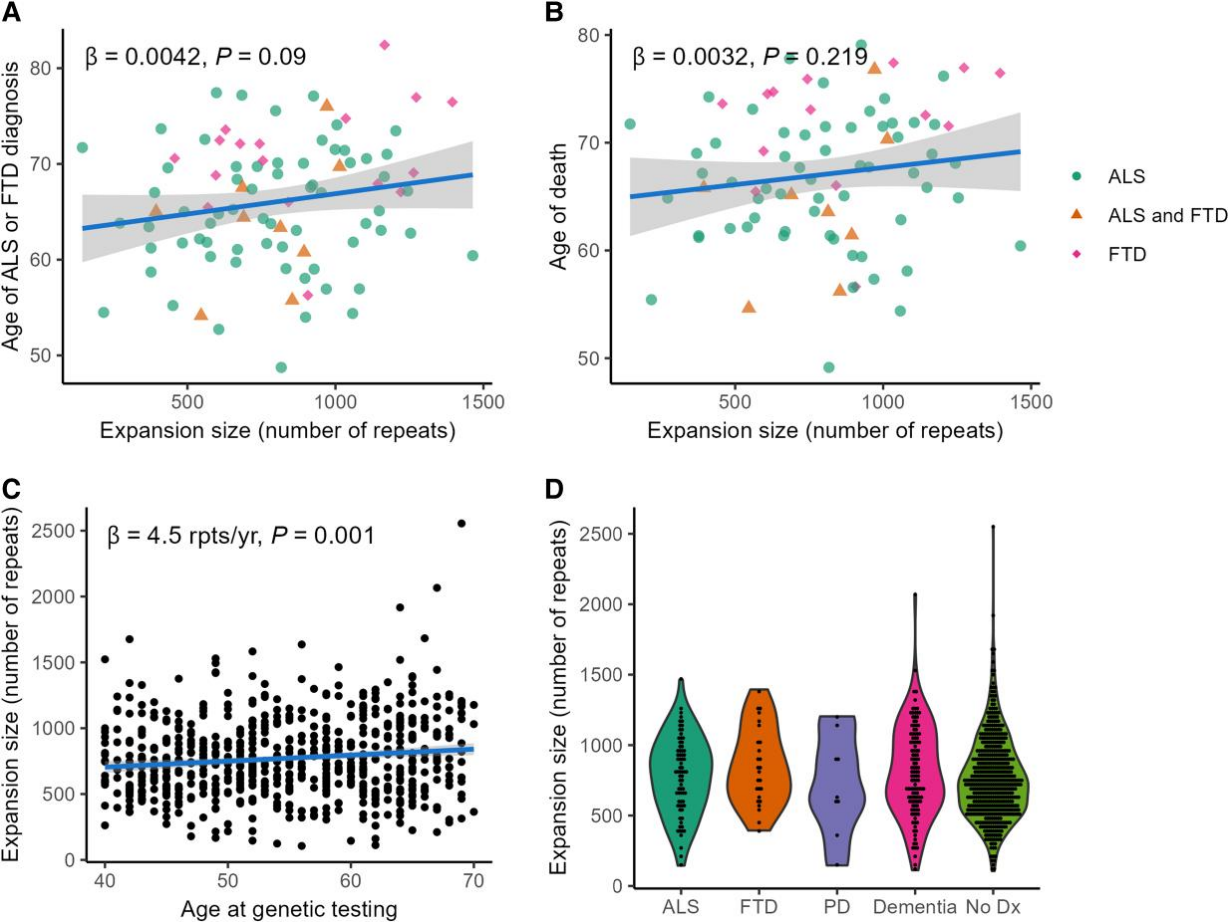
***C9orf72* expansion size and phenotype.** (**A**) Association between *C9orf72* expansion size and age at first recorded ALS or FTD diagnosis (*n* = 91). Points are coloured by diagnosis. (**B**) Association between *C9orf72* expansion size and age of death in individuals with a recorded ALS or FTD diagnosis who have died. Points are coloured by diagnosis (*n* = 81). (**C**) Association between age at genetic testing and expansion size in individuals carrying a *C9orf72* HRE (*n* = 701) (**D**) Expansion size and diagnosis (*n* = 74 for ALS, 26 for FTD, 8 for PD, 121 for any-cause dementia, 521 for No Dx). ALS = amyotrophic lateral sclerosis; FTD = frontotemporal dementia; HRE = hexanucleotide repeat expansion; No Dx = no neurodegenerative diagnosis recorded; PD = parkinsonism. Labels represent fitted linear regression models and associated *P-*values.

*C9orf72* expansion size was not significantly associated with age of first ALS or FTD diagnosis or age of death in people with ALS or FTD but increased with age at study entry in *C9orf72* HRE carriers (beta = 4.5 repeats/year, 95% CI 1.9–5.2, *P* < 0.001; Fig. 2). Expansion size was similar in those with ALS, parkinsonism, dementia and no neurodegenerative diagnosis.

### Effect of UNC13A genotype

**Figure 3:**
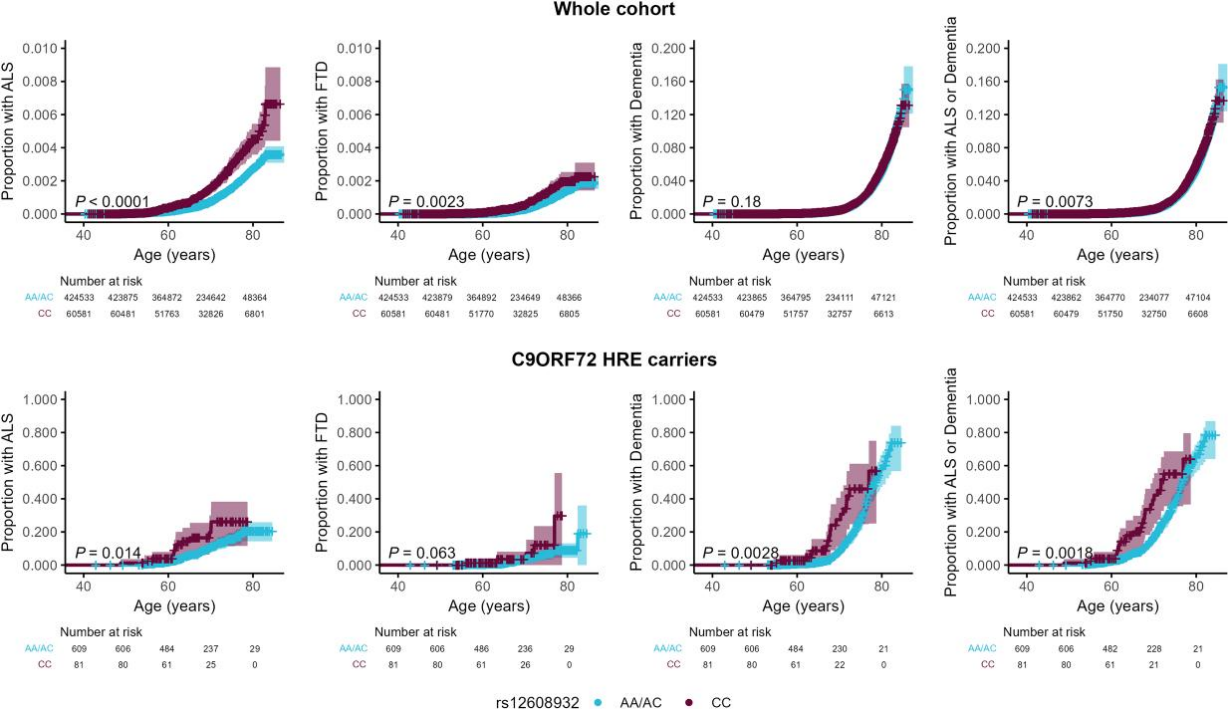
***UNC13A* genotype and neurodegenerative disease incidence.** Kaplan–Meier plots of cumulative incidences of ALS, FTD, dementia and a combined outcome of ALS and dementia by age in individuals stratified by rs12608932 genotype, in the whole UK biobank cohort (*top*, *n* = 485 114) and in individuals carrying the *C9orf72* HRE (*bottom*, *n* = 690). Shaded bands indicate 95% confidence intervals. *P*-values were calculated by the log-rank test. Tables below represent the number of individuals at risk at a given time point. ALS = amyotrophic lateral sclerosis; FTD = frontotemporal dementia; HRE = hexanucleotide repeat expansion.

Among UK Biobank participants with valid genotyping data, homozygosity for the C allele of rs12608932 in *UNC13A* (*n* = 60 581/485 114) was associated with higher risk of ALS, FTD and the combined outcome of ALS or any-cause dementia, compared with other genotypes [ALS: *P*_log-rank_ < 0.001, HR = 1.81 (1.50–2.19); FTD: *P*_log-rank_ = 0.002, HR = 1.58 (1.18–2.13); ALS or dementia: *P*_log-rank_ = 0.007, HR = 1.08 (1.02–1.15); Fig. 3 and Supplementary Tables 8 and 9]. No significant association was observed for any-cause dementia alone [*P*_log-rank_ = 0.18, HR = 1.04 (0.98–1.11)]. A/C heterozygosity did not differ in risk from A/A homozygosity for any outcome (Supplementary Table 8).

In carriers of the *C9orf72* HRE, the CC genotype at rs12608932 (*n* = 81/690) was associated with significantly higher risk of all four outcomes: ALS [*P*_log-rank_ = 0.038, HR = 2.00 (1.09–3.66)], FTD [*P*_log-rank_ = 0.063, HR = 2.03 (1.10–3.73)], dementia [*P*_log-rank_ = 0.011, HR = 2.11 (1.24–3.58)] and ALS or dementia [*P*_log-rank_ = 0.008, HR =1.85 (1.20–2.85)], although for FTD only Cox analyses reached statistical significance, likely due to lower power. The effect sizes for ALS and FTD in *C9orf72* HRE carriers were comparable to those observed in the full cohort. Accordingly, models exploring an interaction between *C9orf72* HRE status and *UNC13A* genotype on risk indicated a potential interaction between genotypes for risk of any-cause dementia and ALS or any-cause dementia, but not ALS or FTD alone (Supplementary Table 10).

## Discussion

In a population-based cohort of 693 people carrying the *C9orf72* HRE, the cumulative incidence of ALS and dementia by age 80 was 66% in those surviving to this age, or 58% accounting for other-cause mortality. No robust association with parkinsonism incidence was detected. ALS incidence peaked between ages 65–75 at around 1% per year, whereas dementia diagnoses were greatest after age 70 at around 5% per year. Additionally, the prevalence of *C9orf72* HRE in this study was 143 per 100 000 (1 in 699), consistent with existing studies suggesting 1 in 517–699.^1,9^ Risk of ALS, FTD and any-cause dementia in *C9orf72* HRE carriers was approximately doubled by the homozygosity for the C allele of rs12608932 in *UNC13A*.

These estimates improve upon previous data by utilizing a prospective population-based cohort design, placing the penetrance of neurodegenerative disease in *C9orf72* HRE carriers lower than estimates from disease-enriched cohorts (>95% by age 80),^1^  ^,13^ but higher than indirect estimates (24%–33%).^14,15^ This further emphasizes the incomplete penetrance of *C9orf72* HRE-associated disease, raising interesting aetiological questions regarding the genetic and environmental factors determining the individualized risk profile of each *C9orf72* HRE carrier. These factors must be carefully considered when counselling asymptomatic individuals considering predictive genetic testing.

In particular, common variants in *UNC13A*—most notably the C allele of rs12608932—have been associated with higher ALS and FTD risk in genome-wide association studies^16,17^ and shorter survival in ALS cohorts.^19^ Mechanistic studies have persuasively linked TDP-43 proteinopathies to *UNC13A* loss of function through cryptic exon inclusion, potentiated by the rs12608932 risk C allele.^18^ An antisense oligonucleotide therapy targeting *UNC13A* as a means to slow disease progression in symptomatic ALS is now also in clinical trials.^22^ This study adds the crucial link between *UNC13A* and *C9orf72* HRE-associated disease in a population-based cohort. These genes appear to exert a proportional effect on risks of ALS and FTD, with *UNC13A* rs12608932 C-allele homozygosity doubling risk in both *C9orf72* HRE carriers and non-carriers. In contrast, for any-cause dementia, there is evidence of a statistical interaction between genotypes, which likely reflects the enrichment of TDP-43-related dementias in those with either *C9orf72* HRE or *UNC13A* risk genotypes compared with the general population, rather than a true synergistic effect.

*UNC13A* genotype may partially explain inter-familial differences in penetrance and inter-individual differences in disease risk,^14^ inform the design of future clinical trials and disease models,^23^ and suggest a targetable pathway for future preventative therapies in *C9orf72* HRE carriers. Further work is required to explore its role in other genetic forms of ALS and FTD.

The finding of a high incidence of recorded non-FTD dementia in *C9orf72* HRE carriers adds to the debate surrounding the spectrum of *C9orf72* HRE-associated disease.^24^ These cases may represent misdiagnosis or mis-recording of FTD.^25^ Alternatively, they may suggest an association between *C9orf72* HRE and amnestic dementias, such as limbic-predominant age-related TDP-43 encephalopathy (LATE) or Alzheimer’s disease, hitherto under-detected due to the skewed selection of individuals for genetic testing. Against the latter possibility is the lack of genetic evidence linking *C9orf72* to dementia in genome-wide association studies.^26^ Adequately resolving these uncertainties requires further study of the neuropsychological and neuropathological phenotypes of these individuals, including analysis of post-mortem tissue.

To reduce the risk of false positive detection given the low prior probability of carrying a *C9orf72* HRE in a population-based cohort, a conservative threshold of 100 repeats was applied for the primary analysis. Analyses of individuals with 30–100 repeats demonstrated a lower incidence of ALS and dementia compared with people with over 100 repeats, which could indicate lower penetrance at shorter repeat lengths. However, given that ExpansionHunter has a reported specificity of 99.9%,^21^ a significant proportion of false positives must be expected, preventing conclusions in this population-based analysis. It is also notable that no association between expansion length and risk of ALS or dementia is observed above 100 repeats.

This study has several limitations. Despite a median follow-up of over 13 years, data are available for fewer individuals at older ages, leading to greater uncertainty in incidence estimates at these ages. Moreover, the UK Biobank cohort is subject to healthy volunteer bias,^27^ the recruitment age (40–69) effectively excludes younger onset disease, and hospital and death registry diagnoses likely significantly post-date symptom onset. The methodology for obtaining diagnostic data in UK Biobank is imperfect and both false positive and false negative cases are likely. Any-cause dementia diagnoses from hospital and mortality data have an estimated positive predictive value of 84.5% (95% CI 74.5–88.8), leading to potential false positive diagnoses and an overestimate of cumulative incidence.^25^ Adjusting for this would reduce the estimated cumulative incidence of any-cause dementia by age 80 to 50% as opposed to the 59% presented. False negatives are difficult to estimate, but the reported dementia incidence of 5.4% by age 80 is far lower than population estimates of around 11%,^28^ though similar to other studies in UK Biobank.^29^ In contrast, ALS diagnoses are generally believed accurate.^30^

### Conclusions

Analysis of *C9orf72* HRE-related disease in a population-based cohort is consistent with incomplete penetrance, with a large proportion manifesting as late-onset dementia. Risk of ALS and dementia in *C9orf72* HRE carriers is influenced by common variants in *UNC13A.* This has implications for genetic counselling and modelling of expected phenoconversion in future preventative trials.

## Supplementary Material

awaf269_Supplementary_Data

## Data Availability

The data that support the findings of this study are available from UK Biobank, subject to a registration and application process (see https://www.ukbiobank.ac.uk).
