# Supplementary material for: Neurodegenerative disease in C9orf72 repeat expansion carriers: population risk and effect of UNC13A
Source: Brain. 2025 Jul 19;148(11):3865–71. doi: 10.1093/brain/awaf269 (PMC12588677; doi:10.1093/brain/awaf269)
Supplement: awaf269_Supplementary_Data [file awaf269_supplementary_data.pdf]

NEURODEGENERATIVE DISEASE INCIDENCE IN *C9ORF72* REPEAT EXPANSION CARRIERS FROM A POPULATION COHORT

SUPPLEMENTARY INFORMATION

Jiali Gao, MB BChir,[1] Andrew G L Douglas, DPhil,[1,2] Christos V. Chalitsios, PhD,[1] Jakub Scaber, DPhil,[1] Kevin Talbot, DPhil,[1] Martin R Turner, PhD,[1] Alexander G Thompson, DPhil[1+]

[1] Nuffield Department of Clinical Neurosciences, University of Oxford, Oxford, UK

[2] Oxford Centre for Genomic Medicine, Oxford University Hospitals NHS Foundation Trust, Oxford, UK

CONTENTS

Supplementary Methods 3

ExpansionHunter 3

Genotyping 3

Neurodegenerative disease outcomes 4

Matching 4

Supplementary Table 1 - UK Biobank Data fields used in this study 6

Supplementary Table 2 – Baseline characteristics of participants carrying *C9ORF72* HRE compared to unmatched non-carriers 7

Supplementary Table 3 – Baseline characteristics of participants carrying *C9ORF72* HRE compared to age, sex, TDI and ethnicity matched non-carriers 9

Supplementary Table 4 – Age-related cumulative incidences of ALS and dementias in carriers of the *C9ORF72* HRE compared to matched controls, using competing risk methods 11

Supplementary Table 5 – Age-related cumulative incidences of ALS and dementias in carriers of the *C9ORF72* HRE compared to matched controls, split by data source, using Kaplan-Meier methods 12

Supplementary Table 6 – Age-related cumulative incidences of ALS and dementias in carriers of the 30-100 repeats in *C9ORF72* compared to matched controls, using Kaplan-Meier methods 13

Supplementary Table 7 – Age-related cumulative incidences of ALS and dementias in carriers of >30 repeats in *C9ORF72* compared to matched controls, using Kaplan-Meier methods 13

Supplementary Table 8 – Age-related cumulative incidences of ALS and dementia by *UNC13A* genotype, using Kaplan-Meier methods 14

Supplementary Table 9 – Hazard ratios of ALS and dementia by *UNC13A* genotype, using Cox proportional Hazard models 16

Supplementary Table 10 – Interaction between *UNC13A* genotype and *C9ORF72* HRE status, using Cox proportional Hazard models 16

Supplementary Figure 1 - Age-related cumulative incidences of ALS and dementias in carriers of the C9ORF72 HRE compared to matched controls, using competing risk methods 17

Supplementary Figure 2– Age-related cumulative incidences of parkinsonism in carriers of the *C9ORF72* HRE compared to matched controls 18

Supplementary Figure 3 – Age of first recorded diagnosis in *C9ORF72* HRE carriers who develop ALS, dementia and parkinsonism compared to matched non-carriers who develop ALS, dementia and parkinsonism 18

Supplementary Figure 4 – Age-related cumulative incidences of ALS and dementias in carriers of the *C9ORF72* HRE compared to matched controls, stratified by sex, BMI, alcohol and smoking categories 19

Supplementary Figure 5 – Age-related cumulative incidences of ALS and dementias in carriers of the *C9ORF72* HRE compared to matched controls by data source 20

Supplementary Figure 6 - Age-related cumulative incidences of ALS and dementias in carriers of 30-100 repeats in *C9ORF72* compared to matched controls, using Kaplan-Meier models 21

Supplementary Figure 7 - Age-related cumulative incidences of ALS and dementias in carriers of >30 repeats in *C9ORF72* compared to matched controls, using Kaplan-Meier models 22

Supplementary Figure 8 – Love plots of covariate balance before and after matching 23

Supplementary Figure 8 – Love plots of covariate balance before and after matching (cont) 24

Supplementary Figure 9 – Density plots of covariate balance before and after matching in *C9ORF72* HRE carriers and controls for cumulative incidence calculations (Match 1) 25

Supplementary Figure 10 – Density plots of covariate balance before and after matching in *C9ORF72* HRE and non-*C9ORF72* ALS patients for age of onset comparison (Match 2) 26

Supplementary Figure 11 – Density plots of covariate balance before and after matching in *C9ORF72* HRE and non-*C9ORF72* dementia patients for age of onset comparison (Match 3) 26

Supplementary Figure 12 – Density plots of covariate balance before and after matching in *C9ORF72* HRE and non-*C9ORF72* parkinsonism patients for age of onset comparison (Match 4) 27

Supplementary Figure 13 – Density plots of covariate balance before and after matching in those with 30-100 repeats in C9ORF72 and controls for cumulative incidence calculations (Match 5) 28

Supplementary Figure 14 – Density plots of covariate balance before and after matching in those with >30 repeats in C9ORF72 and controls for cumulative incidence calculations (Match 6) 29

References 30

STROBE Statement—checklist of items that should be included in reports of observational studies 31

Supplementary Methods

ExpansionHunter

Whole genome sequencing was performed on blood samples from 490,640 participants using Illumina NovaSeq technology,[1] from which *C9ORF72* HRE carriers were identified using ExpansionHunter.[2] This method has previously been shown to detect *C9ORF72* HRE with a sensitivity of 100% and specificity of 99.8-99.9% compared to repeat-primed PCR. ExpansionHunter was run as part of the Illumina DRAGEN (Dynamic Read Analysis for GENomics) 3.7.8 pipeline using the default variant catalogue,[3] which includes 29 "off-target regions" for *C9ORF72* HRE. These represent loci where paired intra-repeat reads may misalign. Their inclusion allows more accurate estimation of repeats larger than the fragment length, but with added risk of overestimating some genotypes.[2,4] Individuals in whom the lower bound of the 95% confidence interval of *C9ORF72* repeat size was over 100 repeats were classed as *C9ORF72* HRE carriers.

In a random sample of 20% of the detected expansion carriers, *C9ORF72* repeat size was also estimated using ExpansionHunter v5 without inclusion of off-target regions and read visualisations of these results were generated using REViewer.[5] These results were all consistent with a repeat size of over at least 30 repeats.

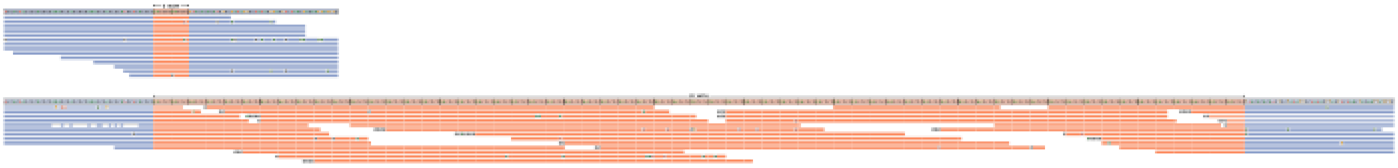

Example read visualisation

As a sensitivity analysis, analyses were repeated in individuals in whom the lower bound of the 95% confidence interval of *C9ORF72* repeat size was over 30 repeats, the more commonly used pathogenicity threshold. However, given the low prior probability of carrying a *C9ORF72* repeat expansion in a population-based cohort and the unavoidable margin of error in estimating repeat length using a computational method, there is a higher risk of false positive classifications in this group.

Genotyping

Genotyping was performed on 488,000 participants using two closely related purpose-designed arrays. ~50,000 participants were run on the UK BiLEVE Axiom array and the remaining ~450,000 were run on the UK Biobank Axiom array.

The genotype of the single nucleotide polymorphism (SNP) rs12608932 in *UNC13A* was extracted from this data. The closely related SNP rs1297319 in *UNC13A,* which has also been linked to ALS/FTD,[6] was not included in these arrays. Given the high degree of linkage disequilibrium between these SNPs ($R^2 = 0.9536$ using LDlink[7]), imputation of this SNP was considered unlikely to add meaningful information.

For quality control, individuals with mismatch between genetically determined and self-reported sex and individuals with sex chromosome aneuploidy were excluded.

Neurodegenerative disease outcomes

Incident neurodegenerative disease diagnoses were identified using the following International Classification of Diseases codes:

All cause dementia:

ICD 9 290.2, 290.3, 290.4, 291.2, 294.1, 331.0, 331.1, 331.2, 331.5

ICD 10 A81.0, F00, F00.0, F00.1, F00.2, F00.9, F01, F01.0, F01.1, F01.2, F01.3, F01.8, F01.9, F02, F02.0, F02.1, F02.2, F02.3, F02.4, F02.8, F03, F05.1, F10.6, G30, G30.0, G30.1, G30.8, G30.9, G31.0, G31.1, G31.8

Frontotemporal dementia:

ICD 9 331.1

ICD 10 F02.0, G31.0

Amyotrophic lateral sclerosis

ICD 9 335.2

ICD 10 G12.2

All cause parkinsonism

ICD 9 3320, 3321, 3330

ICD 10 G20, G21, G21.0, G21.1, G21.2, G21.3, G21.4, G21.8, G21.9, G22, G23.0, G23.1, G23.2, G23.3, G23.8, G23.9

Matching

Nearest neighbour matching by Mahalanobis distance without replacement was used to select suitable controls for four different matched cohorts, labelled Match 1-4. Mahalanobis distance was chosen over propensity score as the distance metric as it resulted in more exact balance of each covariate. After matching, standardised mean differences for all covariates were below 0.1 and Love plots and density plots indicated excellent balance (**Supplementary Figures 8-14**).

Match 1 – For each of 693 *C9ORF72* HRE carriers with complete matching variable data and without prevalent neurodegenerative disease at recruitment, ten controls with fewer than 30 *C9ORF72* repeats, matched by age, sex, ethnicity and Townsend deprivation index, were selected, totalling 6930 controls out of a possible 485,111. This cohort was used to assess the cumulative incidence of neurodegenerative disease by age in *C9ORF72* HRE carriers compared to controls.

Match 2 – For each of 72 *C9ORF72* HRE carriers with an incident diagnosis of ALS, one non-*C9ORF72* HRE carrier with an incident diagnosis of ALS, matched by age and sex, was selected, totalling 72 controls out of a possible 509. This cohort was used for comparison of age of diagnosis in *C9ORF72* HRE versus non-*C9ORF72* ALS cases. The 1:1 ratio was chosen due to the lower number of potential controls available and because higher ratios resulted in poorer balance.

Match 3 – For each of 120 *C9ORF72* HRE carriers with an incident diagnosis of dementia, one non-*C9ORF72* HRE carrier with an incident diagnosis of dementia, matched by age and sex, was selected, totalling 120 controls out of a possible 9114. This cohort was used for comparison of age of diagnosis in *C9ORF72* HRE versus non-*C9ORF72* dementia cases.

Match 4 – For each of 8 *C9ORF72* HRE carriers with an incident diagnosis of parkinsonism, one non-*C9ORF72* HRE carrier with an incident diagnosis of parkinsonism, matched by age and sex, was selected, totalling 8 controls out of a possible 3799. This cohort was used for comparison of age of diagnosis in *C9ORF72* HRE versus non-*C9ORF72* parkinsonism cases.

Match 5 – For each of 110 carriers of 30-100 repeats in *C9ORF72* with complete matching variable data and without prevalent neurodegenerative disease at recruitment, ten controls with fewer than 30 *C9ORF72* repeats, matched by age, sex, ethnicity and Townsend deprivation index, were selected, totalling 1100 controls out of a possible 485,111. This cohort was used to assess the cumulative incidence of neurodegenerative disease by age in carriers of 30-100 repeats in *C9ORF72* compared to controls.

Match 6 – For each of 803 carriers of >30 repeats in *C9ORF72* with complete matching variable data and without prevalent neurodegenerative disease at recruitment, ten controls with fewer than 30 *C9ORF72* repeats, matched by age, sex, ethnicity and Townsend deprivation index, were selected, totalling 8030 controls out of a possible 485,111. This cohort was used to assess the cumulative incidence of neurodegenerative disease by age in carriers of >30 repeats in *C9ORF72* compared to controls.

Analyses were performed using R version 4.3.1.

Supplementary Table 1 - UK Biobank Data fields used in this study

Variable	UKB Field ID*	Category	Notes
Whole genome STR call files (DRAGEN) [500k release]	24062	Exposure	Extraction of *C9ORF72* HRE status
Genotype results	100315	Exposure	Extraction of rs12608932 genotype
Year of birth	34		
Month of birth	52		
Age at recruitment	21022	Exposure, baseline characteristics,matching, covariate in cox analyses	
Sex	31	Baseline characteristics, matching, stratification, covariate in cox analyses	
Ethnic background	21000	Baseline characteristics and matching	
Body mass index (BMI)	21001	Baseline characteristics and stratification	
Smoking status	20116	Baseline characteristics and stratification	
Alcohol intake frequency	1558	Baseline characteristics and stratification	
IPAQ activity group	22032	Baseline characteristics	
Townsend deprivation index at recruitment	22189	Baseline characteristics,matching, covariate in cox analyses	
Age completed full time education	845	Baseline characteristics	
Qualifications	6138	Stratification	
Current employment status	6142	Baseline characteristics	Used if field 20119 is not specified
Current employment status - corrected	20119	Baseline characteristics	
Overall health rating	2178	Baseline characteristics	
Genetic principal components	22009	Covariate in cox analyses	
Genetic sex	22001	Exclusion variable	Used in comparison with 31
Genetic aneuploidy	22019	Exclusion variable	
Genotype measurement batch	22000	Clustering variable	
Assessment centre	54	Clustering variable	
Date of motor neurone disease report	42028	Outcome	
Source of motor neurone disease report	42029	Outcome	
Date of all cause dementia report	42018	Outcome	
Source of all cause dementia report	42019	Outcome	
Date of frontotemporal dementia report	42024	Outcome	
Source of frontotemporal dementia report	42025	Outcome	
Date of all cause parkinsonism report	42030	Outcome	
Source of all cause parkinsonism report	42031	Outcome	
Date lost to follow-up	191	Censoring	
Date of death	NA	Censoring	

*Further details on UKB data fields can be found at https://biobank.ndph.ox.ac.uk/showcase/index.cgi

Supplementary Table 2 – Baseline characteristics of participants carrying *C9ORF72* HRE compared to unmatched non-carriers

	C9ORF72 HRE carriers (N=698)	Non-carriers (N=488025)	Overall (N=488723)	Adjusted P Value
Follow-up time (years)				
Median [IQR]	13.4 [12.3, 14.1]	13.7 [12.9, 14.3]	13.6 [12.9, 14.3]	
Incident ALS diagnosis	72 (10.3 %)	581 (0.1 %)	653 (0.1 %)	
Incident FTD diagnosis	26 (3.7 %)	273 (0.1 %)	299 (0.1 %)	
Incident Dementia diagnosis	120 (17.2 %)	9114 (1.9 %)	9234 (1.9 %)	
Age at recruitment (yrs)				
Median [IQR]	55.0 [48.0, 62.0]	58.0 [50.0, 63.0]	58.0 [50.0, 63.0]	<0.001
Sex				
Male	302 (43.3 %)	223148 (45.7 %)	223450 (45.7 %)	
Female	396 (56.7 %)	264877 (54.3 %)	265273 (54.3 %)	1
Ethnicity				
Non-white	12 (1.7 %)	25842 (5.3 %)	25854 (5.3 %)	
White	683 (97.9 %)	459868 (94.2 %)	460551 (94.2 %)	<0.001
Body Mass Index (kg/m2)				
Median [IQR]	25.3 [23.0, 28.7]	26.7 [24.1, 29.9]	26.7 [24.1, 29.9]	<0.001
Smoking status				
Never	427 (61.2 %)	265712 (54.4 %)	266139 (54.5 %)	
Previous	209 (29.9 %)	168440 (34.5 %)	168649 (34.5 %)	0.011
Current	57 (8.2 %)	51372 (10.5 %)	51429 (10.5 %)	
Alcohol consumption frequency				
Never	71 (10.2 %)	39051 (8.0 %)	39122 (8.0 %)	
<3 times/week	373 (53.4 %)	235939 (48.3 %)	236312 (48.4 %)	0.003
3+ times/week	252 (36.1 %)	211942 (43.4 %)	212194 (43.4 %)	
Physical activity (IPAQ) category				
Low	113 (16.2 %)	69385 (14.2 %)	69498 (14.2 %)	
Moderate	205 (29.4 %)	152523 (31.3 %)	152728 (31.3 %)	1
High	217 (31.1 %)	153370 (31.4 %)	153587 (31.4 %)	
Townsend Deprivation Index				
Median [IQR]	-2.00 [-3.59, 0.768]	-2.15 [-3.65, 0.530]	-2.15 [-3.65, 0.530]	1
Age completed full time education (yrs)				
Median [IQR]	18.0 [16.0, 21.0]	18.0 [16.0, 21.0]	18.0 [16.0, 21.0]	1
Employment status				
Employed	417 (59.7 %)	279596 (57.3 %)	280013 (57.3 %)	1
Not employed	280 (40.1 %)	206442 (42.3 %)	206722 (42.3 %)	
Self-rated overall health				

	C9ORF72 HRE carriers (N=698)	Non-carriers (N=488025)	Overall (N=488723)	Adjusted P Value
Excellent	122 (17.5 %)	80102 (16.4 %)	80224 (16.4 %)	1
Good	422 (60.5 %)	281736 (57.7 %)	282158 (57.7 %)	
Fair	131 (18.8 %)	101726 (20.8 %)	101857 (20.8 %)	
Poor	21 (3.0 %)	21493 (4.4 %)	21514 (4.4 %)	
rs12608932 genotype				
AA	292 (41.8%)	202184 (41.4 %)	202476 (41.4 %)	1
AC	317 (45.4%)	219668 (45.0 %)	219985 (45.0 %)	
CC	82 (11.7%)	60207 (12.3 %)	60289 (12.3 %)	
Not available	7 (1.0%)	5966 (1.2%)	5973 (1.2%)	
Minor allele frequency (%)	*34.8*	*35.3*	*35.3*	

Individuals without whole genome sequencing data and individuals with prevalent neurodegenerative diagnoses (3 out of 701 *C9ORF72* HRE carriers) have been excluded.

C9ORF72 HRE carriers = participants carrying at least one *C9ORF72* HRE allele of over 100 repeats; non-carriers = participants homozygous for unexpanded *C9ORF72* (< 30 repeats); ALS = amyotrophic lateral sclerosis; FTD = frontotemporal dementia; IPAQ = international physical activity questionnaire. Adjusted *p* value represents *p* value from chi squared (for categorical variables) or Mann Whitney U (for continuous variables) tests, adjusted for multiple testing by Bonferroni correction

Supplementary Table 3 – Baseline characteristics of participants carrying *C9ORF72* HRE compared to age, sex, TDI and ethnicity matched non-carriers

	C9ORF72 HRE carriers (N=693)	Matched non-carriers (N=6930)	Adjusted P Value
Follow-up time (years)			
Median [IQR]	13.4 [12.3, 14.1]	13.7 [13.0, 14.3]	
Incident ALS diagnosis	71 (10.2 %)	10 (0.1 %)	
Incident FTD diagnosis	25 (3.6 %)	3 (0.0 %)	
Incident Dementia diagnosis	119 (17.2 %)	104 (1.5 %)	
Age at recruitment (yrs)			
Median [IQR]	55.0 [48.0, 62.0]	55.0 [47.0, 62.0]	
Sex			
Male	298 (43.0 %)	2980 (43.0 %)	
Female	395 (57.0 %)	264877 (54.3 %)	
Ethnicity			
Non-white	12 (1.7 %)	25842 (5.3 %)	
White	681 (98.3 %)	3950 (57.0 %)	
Body Mass Index (kg/m2)			
Median [IQR]	25.3 [22.9, 28.7]	26.6 [24.0, 29.8]	<0.001
Smoking status			
Never	424 (61.2 %)	3846 (55.5 %)	
Previous	208 (30.0 %)	2296 (33.1 %)	0.039
Current	57 (8.2 %)	770 (11.1 %)	
Alcohol consumption frequency			
Never	70 (10.1 %)	477 (6.9 %)	
<3 times/week	370 (53.4 %)	3431 (49.5 %)	<0.001
3+ times/week	252 (36.4 %)	3014 (43.5 %)	
Physical activity (IPAQ) category			
Low	113 (16.3 %)	1001 (14.4 %)	
Moderate	202 (29.1 %)	2143 (30.9 %)	1
High	216 (31.2 %)	2239 (32.3 %)	
Townsend Deprivation Index			
Median [IQR]	-1.99 [-3.58, 0.760]	-1.99 [-3.59, 0.770]	1
Age completed full time education (yrs)			
Median [IQR]	18.0 [16.0, 21.0]	18.0 [16.0, 21.0]	1
Employment status			
Employed	415 (59.9 %)	4331 (62.5 %)	1
Not employed	277 (40.0 %)	2576 (37.2 %)	

	C9ORF72 HRE carriers (N=693)	Matched non-carriers (N=6930)	Adjusted P Value
Self-rated overall health			
Excellent	121 (17.5 %)	1131 (16.3 %)	0.636
Good	421 (60.8 %)	4006 (57.8 %)	
Fair	129 (18.6 %)	1441 (20.8 %)	
Poor	21 (3.0 %)	321 (4.6 %)	
rs12608932 genotype			
AA	290 (41.8 %)	2844 (41.0 %)	1
AC	315 (45.5 %)	3125 (45.1 %)	
CC	82 (11.8 %)	868 (12.5 %)	
Not available	6 (0.9%)	93 (1.3%)	
Minor allele frequency (%)	*34.9*	*35.5*	

Individuals without whole genome sequencing data and individuals with prevalent neurodegenerative diagnoses or with missing data for any of the matching variables (8 out of 701 *C9ORF7* HRE carriers) have been excluded.

C9ORF72 HRE carriers = participants carrying at least one *C9ORF72* HRE allele of over 100 repeats; matched non-carriers = participants homozygous for unexpanded *C9ORF72* (< 30 repeats) matched by age, sex, Townsend deprivation index and ethnicity; ALS = amyotrophic lateral sclerosis; FTD = frontotemporal dementia; IPAQ = international physical activity questionnaire

Adjusted *p* value represents *p* value from chi squared (for categorical variables) or Mann Whitney U (for continuous variables) tests, adjusted for multiple testing by Bonferroni correction

Supplementary Table 4 – Age-related cumulative incidences of ALS and dementias in carriers of the *C9ORF72* HRE compared to matched controls, using competing risk methods

C9ORF72 status	Outcome	Incidence by 65 years, % (95% CI)	Incidence by 70 years, % (95% CI)	Incidence by 75 years, % (95% CI)	Incidence by 80 years, % (95% CI)
No C9 exp	ALS	0.04 (0.01 - 0.13)	0.04 (0.01 - 0.13)	0.27 (0.13 - 0.54)	0.38 (0.19 - 0.70)
C9 exp	ALS	6.3 (4.5 - 8.6)	11 (8.4 – 14)	15 (12 – 19)	18 (14 – 22)
No C9 exp	FTD	0.04 (0.01 - 0.15)	0.04 (0.01 - 0.15)	0.08 (0.02 - 0.22)	0.08 (<0.02 - 0.22)
C9 exp	FTD	0.9 (0.3 - 2.0)	2.9 (1.6 - 4.9)	5.7 (3.6 - 8.5)	7.3 (4.7 – 11)
No C9 exp	Dementia	0.15 (0.07 - 0.29)	0.35 (0.21 - 0.57)	1.3 (0.95 - 1.8)	4.8 (3.8 - 6.0)
C9 exp	Dementia	2.2 (1.2 - 3.7)	8.9 (6.3 – 12)	24 (19 – 29)	44 (37 – 51)
No C9 exp	ALS or Dementia	0.16 (0.08 - 0.31)	0.37 (0.22 - 0.59)	1.6 (1.2 -2.1)	5.1 (4.1 - 6.4)
C9 exp	ALS or Dementia	7.6 (5.5 – 10)	18 (15 – 22)	36 (31 – 41)	58 (50 – 64)
No C9 exp	Parkinsonism	0.13 (0.06 - 0.26)	0.24 (0.12 - 0.42)	0.88 (0.60 - 1.3)	2.1 (1.5 - 2.9)
C9 exp	Parkinsonism	0.33 (0.07 - 1.1)	0.60 (0.16 - 1.7)	2.1 (0.90 - 4.3)	2.7 (1.2 - 5.3)

C9 exp = participants carrying at least one C9ORF72 HRE of over 100 repeats (n =693); No C9 exp = participants homozygous for unexpanded C9ORF72 (< 30 repeats) matched by age, sex, Townsend deprivation index and ethnicity (n= 6930); ALS = amyotrophic lateral sclerosis; FTD = frontotemporal dementia. Numbers rounded to 2 significant figures.

Supplementary Table 5 – Age-related cumulative incidences of ALS and dementias in carriers of the *C9ORF72* HRE compared to matched controls, split by data source, using Kaplan-Meier methods

C9ORF72 status	Outcome – data source	Incidence by 65 years, % (95% CI)	Incidence by 70 years, % (95% CI)	Incidence by 75 years, % (95% CI)	Incidence by 80 years, % (95% CI)
No C9 exp	ALS – HES	<0.1 (0 - <0.1)	<0.1 (0 - <0.1)	0.3 (<0.1 - 0.5)	0.4 (0.1 - 0.7)
	ALS – death	<0.1 (0 - <0.1)	<0.1 (0 - <0.1)	<0.1 (0 - 0.2)	0.3 (<0.1 - 0.5)
	ALS – all data	<0.1 (0 - <0.1)	<0.1 (0 - <0.1)	0.3 (<0.1 - 0.5)	0.4 (0.1 - 0.7)
C9 exp	ALS – HES	5.9 (3.9 - 7.9)	11 (7.8 - 14)	16 (12 - 20)	20 (15 - 25)
	ALS – death	4.3 (2.6 - 6.1)	9.3 (6.4 - 12)	15 (11 - 19)	20 (13 - 26)
	ALS – all data	6.5 (4.3 - 8.5)	12 (8.4 – 15)	17 (13 – 21)	21 (16 – 27)
No C9 exp	FTD – HES	<0.1 (0 - <0.1)	<0.1 (0 - <0.1)	<0.1 (0 - 0.1)	<0.1 (0 - 0.1)
	FTD – death	<0.1 (0 - <0.1)	<0.1 (0 - 0.1)	<0.1 (0 - 0.1)	<0.1 (0 - 0.1)
	FTD – all data	<0.1 (0 - 0.1)	<0.1 (0 - 0.1)	<0.1 (0 - 0.2)	<0.1 (0 - 0.2)
C9 exp	FTD – HES	0.9 (0.1 - 1.8)	2.7 (1.1 - 4.4)	6.6 (3.4 - 9.7)	7.7 (3.9 - 11)
	FTD – death	0.6 (0 - 1.2)	1.4 (0.2 - 2.6)	3.2 (0.9 - 5.5)	8.4 (3.3 - 13)
	FTD – all data	0.9 (0.1 - 1.8)	3.2 (1.4 - 5.0)	7.1 (3.9 – 10)	10 (5.4 – 15)
No C9 exp	Dementia – HES	0.1 (<0.1 - 0.2)	0.3 (0.2 - 0.5)	1.3 (0.9 - 1.8)	5.0 (3.8 - 6.2)
	Dementia – death	<0.1 (0 - <0.1)	<0.1 (0 - 0.2)	0.2 (<0.1 - 0.4)	1.7 (1.0 - 2.5)
	Dementia – all data	0.2 (<0.1 - 0.3)	0.4 (0.2 - 0.5)	1.4 (1.0 - 1.9)	5.4 (4.1 - 6.7)
C9 exp	Dementia – HES	1.9 (0.7 - 3.0)	8.0 (5.1 - 11)	26 (20 - 32)	56 (45 - 64)
	Dementia – death	1.1 (0.2 - 2.1)	4.2 (2.1 - 6.2)	18 (13 - 23)	37 (28 - 45)
	Dementia – all data	2.2 (1.0 - 3.5)	9.8 (6.7 – 13)	29 (23 – 35)	59 (49 – 67)
No C9 exp	ALS or Dementia – HES	0.1 (<0.1 - 0.2)	0.4 (0.2 - 0.5)	1.6 (1.1 - 2.1)	5.4 (4.2 - 6.6)
	ALS or Dementia – death	<0.1 (0 - <0.1)	<0.1 (0 - 0.2)	0.3 (<0.1 - 0.5)	2.0 (1.2 - 2.7)
	ALS or Dementia – all data	0.2 (<0.1 - 0.3)	0.4 (0.2 - 0.6)	1.7 (1.2 - 2.2)	5.8 (4.5 - 7.0)
C9 exp	ALS or Dementia – HES	7.2 (4.9 - 9.3)	17 (13 - 21)	37 (31 - 42)	63 (54 - 71)
	ALS or Dementia – death	4.9 (3.0 - 6.7)	12 (9.0 - 15)	30 (24 - 35)	49 (40 - 56)
	ALS or Dementia – all data	7.7 (5.4 - 9.9)	19 (15 – 23)	39 (33 – 45)	66 (57 – 73)

C9 exp = participants carrying at least one C9ORF72 HRE of over 100 repeats (n =693); No C9 exp = participants homozygous for unexpanded C9ORF72 (< 30 repeats) matched by age, sex, Townsend deprivation index and ethnicity (n= 6930); ALS = amyotrophic lateral sclerosis; FTD = frontotemporal dementia; HES = hospital episode statistics; death = death certificate data. Numbers rounded to 2 significant figures.

Supplementary Table 6 – Age-related cumulative incidences of ALS and dementias in carriers of the 30-100 repeats in *C9ORF72* compared to matched controls, using Kaplan-Meier methods

C9ORF72 status	Outcome	Incidence by 65 years, % (95% CI)	Incidence by 70 years, % (95% CI)	Incidence by 75 years, % (95% CI)	Incidence by 80 years, % (95% CI)
No C9 exp	ALS	0 (0 - 0)	0 (0 - 0)	0 (0 - 0)	0.5 (0 - 1.5)
C9 exp	ALS	1.3 (0 - 3.7)	2.8 (0 - 6.7)	2.8 (0 - 6.7)	2.8 (0 - 6.7)
No C9 exp	FTD	0.2 (0 - 0.5)	0.2 (0 - 0.5)	0.2 (0 - 0.5)	0.2 (0 - 0.5)
C9 exp	FTD	0 (0 - 0)	0 (0 - 0)	0 (0 - 0)	0 (0 - 0)
No C9 exp	Dementia	0.3 (0 - 0.7)	0.5 (0 - 1.0)	1.5 (0.4 - 2.6)	5.4 (2.4 - 8.2)
C9 exp	Dementia	0 (0 - 0)	1.4 (0 - 4.1)	4.1 (0 - 9.7)	16 (1.2 - 29)
No C9 exp	ALS or Dementia	0.3 (0 - 0.7)	0.5 (0 - 1.0)	1.5 (0.4 - 2.6)	5.9 (2.8 - 8.9)
C9 exp	ALS or Dementia	1.3 (0 - 3.7)	4.2 (0 - 8.8)	6.8 (0 – 13)	19 (3.5 – 31)
No C9 exp	Parkinsonism	0.2 (0 - 0.5)	0.7 (<0.1 - 1.4)	1.4 (0.4 - 2.4)	2.6 (0.9 - 4.4)
C9 exp	Parkinsonism	0 (0 - 0)	0 (0 - 0)	0 (0 - 0)	5.3 (0 – 15)

C9 exp = participants carrying 30-100 repeats in *C9ORF72* (n =110); No C9 exp = participants homozygous for unexpanded C9ORF72 (< 30 repeats) matched by age, sex, Townsend deprivation index and ethnicity (n= 1099); ALS = amyotrophic lateral sclerosis; FTD = frontotemporal dementia. Numbers rounded to 2 significant figures.

Supplementary Table 7 – Age-related cumulative incidences of ALS and dementias in carriers of >30 repeats in *C9ORF72* compared to matched controls, using Kaplan-Meier methods

C9ORF72 status	Outcome	Incidence by 65 years, % (95% CI)	Incidence by 70 years, % (95% CI)	Incidence by 75 years, % (95% CI)	Incidence by 80 years, % (95% CI)
No C9 exp	ALS	<0.1 (0 - <0.1)	<0.1 (0 - <0.1)	0.2 (<0.1 - 0.4)	0.4 (0.1 - 0.7)
C9 exp	ALS	5.7 (3.9 - 7.5)	10 (7.6 – 13)	15 (11 – 18)	18 (14 – 23)
No C9 exp	FTD	<0.1 (<0.1 - 0.1)	<0.1 (<0.1 - 0.1)	<0.1 (<0.1 - 0.2)	<0.1 (<0.1 - 0.2)
C9 exp	FTD	0.8 (<0.1 - 1.5)	2.7 (1.2 - 4.2)	5.9 (3.2 - 8.5)	8.3 (4.5 – 12)
No C9 exp	Dementia	0.2 (<0.1 - 0.3)	0.4 (0.2 - 0.5)	1.4 (1.0 - 1.8)	5.5 (4.3 - 6.6)
C9 exp	Dementia	1.9 (0.8 - 3.0)	8.5 (5.8 – 11)	25 (20 – 30)	51 (42 – 58)
No C9 exp	ALS or Dementia	0.2 (<0.1 - 0.3)	0.4 (0.2 - 0.6)	1.6 (1.2 - 2.1)	5.8 (4.6 - 7.0)
C9 exp	ALS or Dementia	6.8 (4.8 - 8.7)	17 (13 – 20)	34 (29 – 39)	58 (50 – 65)
No C9 exp	Parkinsonism	0.1 (<0.1 - 0.2)	0.3 (0.2 - 0.5)	0.9 (0.6 - 1.3)	2.3 (1.6 - 3.1)
C9 exp	Parkinsonism	0.3 (0 - 0.7)	0.5 (0 - 1.2)	2.5 (0.5 - 4.4)	5.0 (0.9 - 9.0)

C9 exp = participants carrying >30 repeats in *C9ORF72* (n =803); No C9 exp = participants homozygous for unexpanded C9ORF72 (< 30 repeats) matched by age, sex, Townsend deprivation index and ethnicity (n= 8030); ALS = amyotrophic lateral sclerosis; FTD = frontotemporal dementia. Numbers rounded to 2 significant figures.

Supplementary Table 8 – Age-related cumulative incidences of ALS and dementia by *UNC13A* genotype, using Kaplan-Meier methods

Cohort	rs12608932 genotype	Outcome	Incidence by 65 years, % (95% CI)	Incidence by 70 years, % (95% CI)	Incidence by 75 years, % (95% CI)	Incidence by 80 years, % (95% CI)
Whole cohort	AA	ALS	<0.1 (<0.1 - <0.1)	<0.1 (<0.1 - <0.1)	0.2 (0.1 - 0.2)	0.3 (0.3 - 0.3)
Whole cohort	AC	ALS	<0.1 (<0.1 - <0.1)	<0.1 (<0.1 - <0.1)	0.1 (0.1 - 0.2)	0.3 (0.2 - 0.3)
Whole cohort	AA/AC combined	ALS	<0.1 (<0.1 - <0.1)	<0.1 (<0.1 - <0.1)	0.2 (0.1 - 0.2)	0.3 (0.2 - 0.3)
Whole cohort	CC	ALS	<0.1 (<0.1 - <0.1)	0.2 (0.1 - 0.2)	0.3 (0.2 - 0.3)	0.5 (0.4 - 0.5)
Whole cohort	AA	FTD	<0.1 (<0.1 - <0.1)	<0.1 (<0.1 - <0.1)	<0.1 (<0.1 - <0.1)	0.1 (0.1 - 0.2)
Whole cohort	AC	FTD	<0.1 (<0.1 - <0.1)	<0.1 (<0.1 - <0.1)	<0.1 (<0.1 - <0.1)	0.1 (0.1 - 0.2)
Whole cohort	AA/AC combined	FTD	<0.1 (<0.1 - <0.1)	<0.1 (<0.1 - <0.1)	<0.1 (<0.1 - <0.1)	0.1 (0.1 - 0.2)
Whole cohort	CC	FTD	<0.1 (<0.1 - <0.1)	<0.1 (<0.1 - <0.1)	0.1 (<0.1 - 0.2)	0.2 (0.1 - 0.3)
Whole cohort	AA	Dementia	0.1 (0.1 - 0.1)	0.4 (0.4 - 0.5)	1.6 (1.5 - 1.7)	5.1 (4.9 - 5.3)
Whole cohort	AC	Dementia	0.2 (0.1 - 0.2)	0.5 (0.4 - 0.5)	1.7 (1.6 - 1.8)	5.3 (5.1 - 5.5)
Whole cohort	AA/AC combined	Dementia	0.1 (0.1 - 0.2)	0.4 (0.4 - 0.5)	1.6 (1.6 - 1.7)	5.2 (5.0 - 5.3)
Whole cohort	CC	Dementia	0.2 (0.1 - 0.2)	0.4 (0.4 - 0.5)	1.7 (1.6 - 1.9)	5.5 (5.2 - 5.9)
Whole cohort	AA	ALS or Dementia	0.2 (0.1 - 0.2)	0.5 (0.5 - 0.5)	1.7 (1.7 - 1.8)	5.3 (5.1 - 5.5)
Whole cohort	AC	ALS or Dementia	0.2 (0.2 - 0.2)	0.5 (0.5 - 0.6)	1.8 (1.7 - 1.9)	5.5 (5.3 - 5.7)
Whole cohort	AA/AC combined	ALS or Dementia	0.2 (0.2 - 0.2)	0.5 (0.5 - 0.5)	1.8 (1.7 - 1.8)	5.4 (5.3 - 5.6)
Whole cohort	CC	ALS or Dementia	0.2 (0.2 - 0.3)	0.6 (0.5 - 0.6)	2.0 (1.8 - 2.1)	5.9 (5.5 - 6.3)
C9ORF72 carriers	AA	ALS	5.9 (2.8 - 8.8)	12 (7.2 - 17)	16 (10 - 22)	22 (13 - 30)
C9ORF72 carriers	AC	ALS	5.4 (2.5 - 8.3)	9.3 (5.1 - 13)	15 (8.3 - 20)	19 (10 - 26)
C9ORF72 carriers	AA/AC combined	ALS	5.6 (3.5 - 7.7)	11 (7.5 - 14)	15 (11 - 19)	20 (14 - 26)
C9ORF72 carriers	CC	ALS	14 (5.0 - 22)	20 (7.8 - 30)	26 (12 - 38)	Insufficient data
C9ORF72 carriers	AA	FTD	0.9 (0 - 2.1)	3.3 (0.6 - 5.9)	6.9 (2.0 - 12)	8.8 (2.6 - 15)
C9ORF72 carriers	AC	FTD	0.7 (0 - 1.8)	3.5 (0.6 - 6.3)	6.4 (2.0 - 11)	8.7 (2.4 - 15)
C9ORF72 carriers	AA/AC combined	FTD	0.8 (<0.1 - 1.6)	3.4 (1.4 - 5.3)	6.7 (3.4 - 9.9)	8.8 (4.4 - 13)
C9ORF72 carriers	CC	FTD	3.4 (0 - 8.1)	3.4 (0 - 8.1)	12 (0 - 24)	Insufficient data
C9ORF72 carriers	AA	Dementia	1.3 (0 - 2.9)	7.3 (3.1 - 11)	28 (19 - 36)	63 (46 - 75)
C9ORF72 carriers	AC	Dementia	1.6 (<0.1 - 3.2)	8.7 (4.0 - 13)	26 (17 - 34)	53 (38 - 64)

C9ORF72 carriers	AA/AC combined	Dementia	1.5 (0.4 - 2.6)	7.9 (4.9 - 11)	27 (20 - 33)	58 (47 - 66)
C9ORF72 carriers	CC	Dementia	8.6 (0.9 - 16)	27 (11 - 41)	46 (25 - 61)	Insufficient data
C9ORF72 carriers	AA	ALS or Dementia	6.3 (3.1 - 9.4)	16 (11 - 21)	38 (29 - 46)	70 (56 - 80)
C9ORF72 carriers	AC	ALS or Dementia	6.6 (3.4 - 9.7)	17 (11 - 22)	36 (27 - 44)	60 (46 - 70)
C9ORF72 carriers	AA/AC combined	ALS or Dementia	6.4 (4.2 - 8.6)	17 (13 - 20)	37 (31 - 43)	65 (56 - 73)
C9ORF72 carriers	CC	ALS or Dementia	18 (7.5 - 27)	39 (23 - 52)	55 (36 - 69)	Insufficient data

Whole cohort = all participants with valid data in UK Biobank cohort (n =485114); *C9ORF72* carriers = participants carrying > 100 repeats in *C9ORF72* with valid data (n= 690); ALS = amyotrophic lateral sclerosis. Numbers rounded to 2 significant figures

Supplementary Table 9 – Hazard ratios of ALS and dementia by *UNC13A* genotype, using Cox proportional Hazard models

Cohort	Outcome	Hazard ratio (95% CI) for rs12608932 CC genotype compared to AA/AC	
		Model 1	Model 2
Whole cohort	ALS	1.81 (1.50 - 2.19)	1.83 (1.51 - 2.22)
Whole cohort	FTD	1.58 (1.18 - 2.13)	1.59 (1.18 - 2.13)
Whole cohort	Dementia	1.04 (0.98 - 1.11)	1.05 (0.99 - 1.11)
Whole cohort	ALS or Dementia	1.08 (1.02 - 1.15)	1.09 (1.03 - 1.16)
C9ORF72 carriers	ALS	2.00 (1.09 - 3.66)	2.13 (1.15 - 3.93)
C9ORF72 carriers	FTD	2.03 (1.10 - 3.73)	2.62 (0.90 - 7.65)
C9ORF72 carriers	Dementia	2.11 (1.24 - 3.58)	2.08 (1.20 - 3.63)
C9ORF72 carriers	ALS or Dementia	1.85 (1.20 - 2.85)	1.85 (1.19 - 2.88)

Whole cohort = all participants with valid data in UK Biobank cohort (n =485114); *C9ORF72* carriers = participants carrying > 100 repeats in *C9ORF72* with valid data (n= 690); ALS = amyotrophic lateral sclerosis, FTD = frontotemporal dementia. Numbers rounded to 2 decimal places

Hazard ratios are calculated using Cox regression models with age as the timescale. Model 1 (primary analysis) is adjusted for sex, age at recruitment, Townsend deprivation index and with robust standard errors clustered by assessment centre. Model 2 (sensitivity analysis) was additionally adjusted for the first 15 genetic principal components and clustered by genetic batch.

Supplementary Table 10 – Interaction between *UNC13A* genotype and *C9ORF72* HRE status, using Cox proportional Hazard models

Outcome	Hazard ratio for rs12608932 CC vs AA/AC	Hazard ratio for *C9ORF72* HRE status	Hazard ratio for interaction term	P value for interaction term	AIC excluding interaction term	AIC including interaction term
ALS	1.84	122.34	1.24	0.53	15166	15168
FTD	1.56	95.86	1.72	0.32	6875	6876
Dementia	1.04	17.83	2.32	2.80×10^{-8}	210326	210320
ALS or Dementia	1.08	24.71	2.15	5.80×10^{-4}	224381	224372

Interaction models in all participants with valid data in UK Biobank cohort (n =485114); *C9ORF72* HRE status = > 100 repeats vs <100 repeats; ALS = amyotrophic lateral sclerosis, FTD = frontotemporal dementia, AIC = Akaike Information Criterion (lower = better fit). Numbers rounded to 2 decimal places

Hazard ratios are calculated using Cox regression models with age as the timescale, adjusted for sex, age at recruitment, Townsend deprivation index and with robust standard errors clustered by assessment centre.

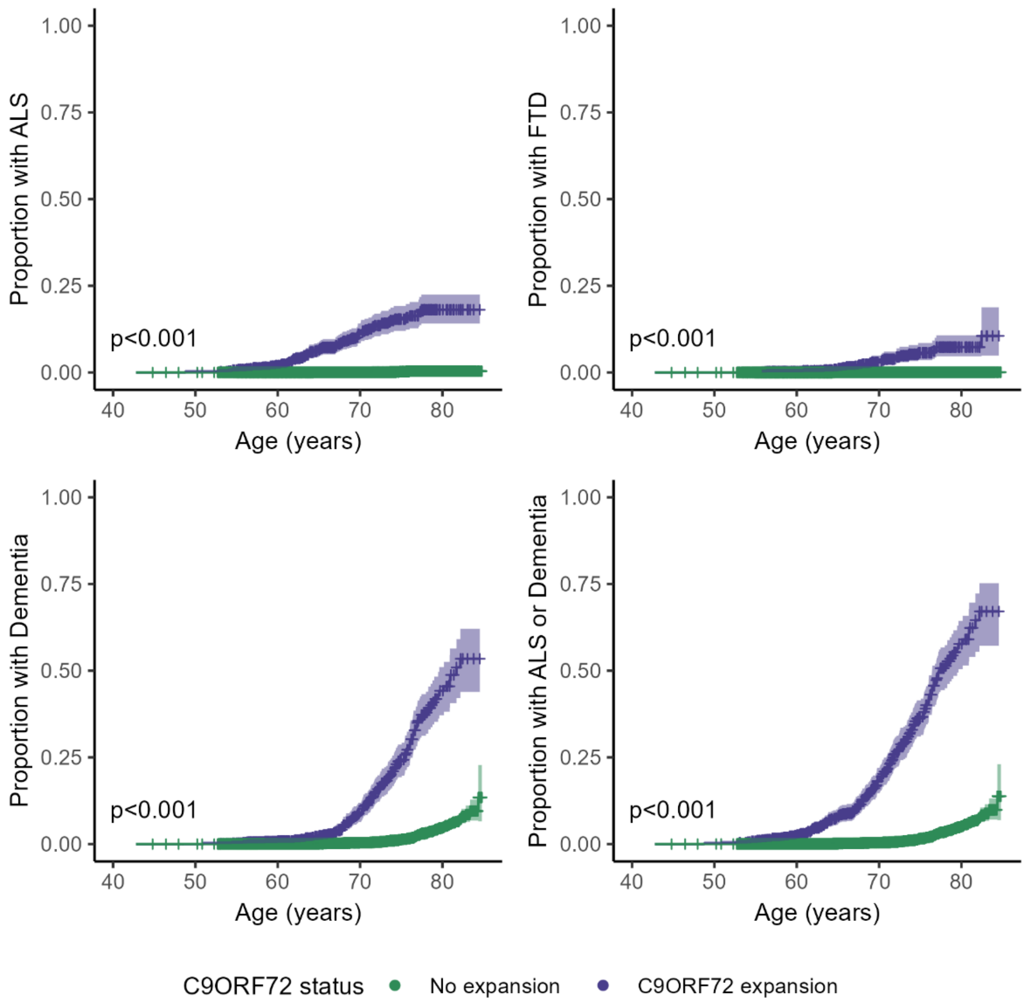

Supplementary Figure 1 - Age-related cumulative incidences of ALS and dementias in carriers of the C9ORF72 HRE compared to matched controls, using competing risk methods

Cumulative incidences of ALS, FTD, dementia and a combined outcome of ALS and dementia by age in individuals carrying the *C9ORF72* HRE (purple, n =693) compared to age, sex, Townsend deprivation index and ethnicity matched controls (green, n= 6930), accounting for the competing risk of death by other causes. Shaded bands indicate 95% confidence intervals.

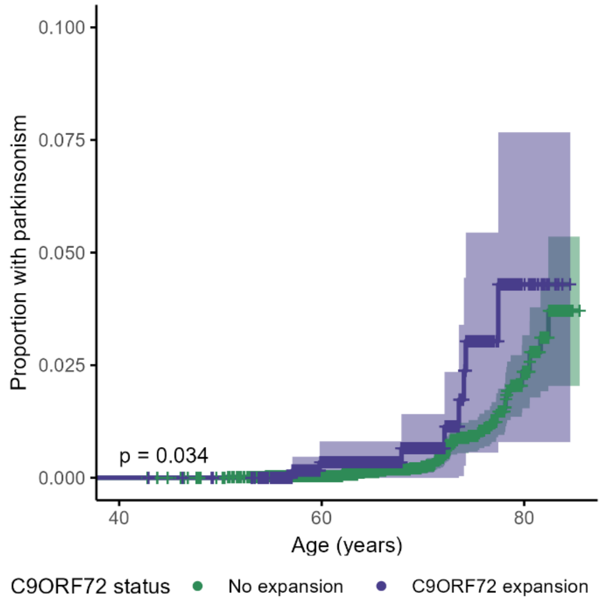

Supplementary Figure 2– Age-related cumulative incidences of parkinsonism in carriers of the *C9ORF72* HRE compared to matched controls

Kaplan-Meier plots of cumulative incidence of parkinsonism by age in individuals carrying the *C9ORF72* HRE (purple, n=693) compared to age, sex, Townsend deprivation index and ethnicity matched controls (green, n=6930). Shaded bands indicate 95% confidence intervals. *P* values were calculated by the log-rank test.

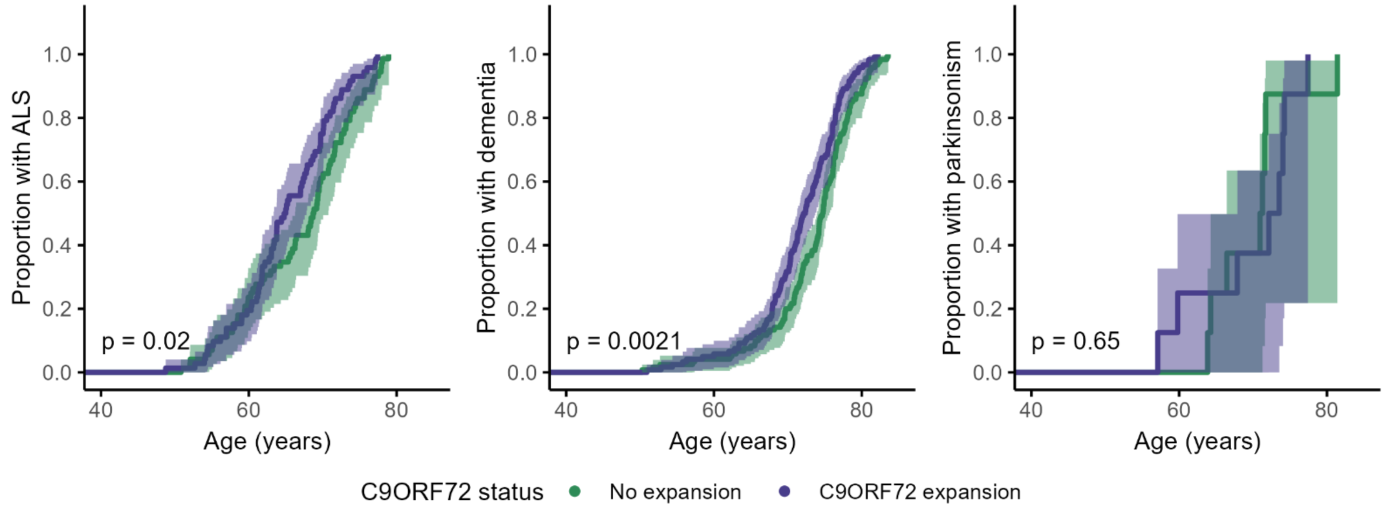

Supplementary Figure 3 – Age of first recorded diagnosis in *C9ORF72* HRE carriers who develop ALS, dementia and parkinsonism compared to matched non-carriers who develop ALS, dementia and parkinsonism

Kaplan-Meier analyses restricted to individuals with a recorded diagnosis of ALS (left), dementia (middle), or parkinsonism (right), comparing cumulative diagnoses by age in individuals carrying a *C9ORF72* HRE (purple, n=72 for ALS, n=120 for dementia, n=8 for parkinsonism) with age and sex-matched ALS/dementia patients who do not carry a *C9ORF72* HRE (green, n=72 for ALS, n=120 for dementia, n=8 for parkinsonism). Shaded bands indicate 95% confidence intervals. *P* values were calculated by the log-rank test.

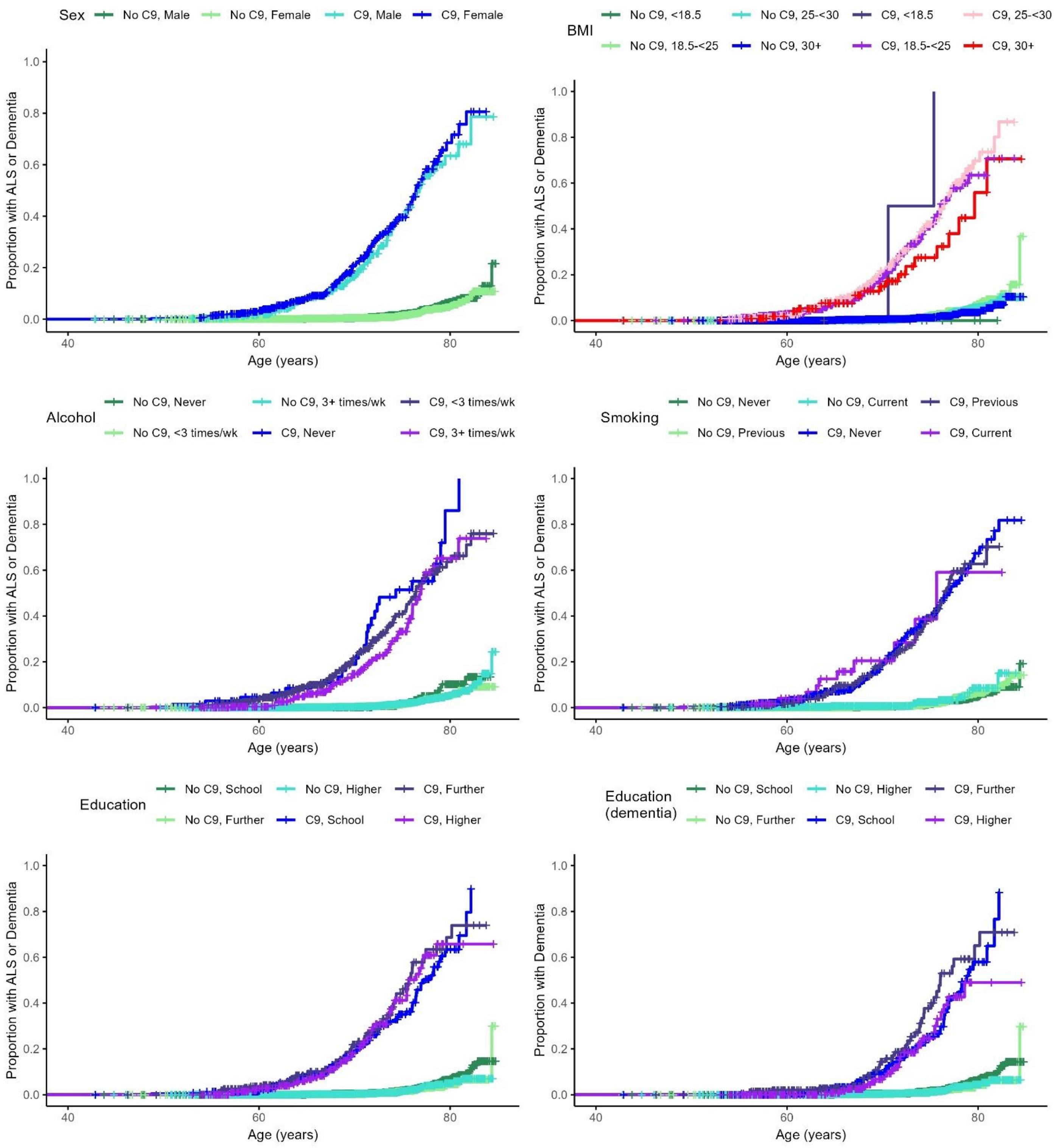

Supplementary Figure 4 – Age-related cumulative incidences of ALS and dementias in carriers of the *C9ORF72* HRE compared to matched controls, stratified by sex, BMI, alcohol,smoking and highest eduation categories

Cumulative incidences of ALS and dementia by age in individuals carrying the *C9ORF72* HRE ("C9", n =693) compared to age, sex, Townsend deprivation index and ethnicity matched controls ("No C9", n= 6930), stratified by sex (male, female; top left), BMI (kg/m^2, top right), alcohol intake frequency (middle left), smoking status (middle right) and highest educational qualification (school leaver, further education or higher education; bottom).

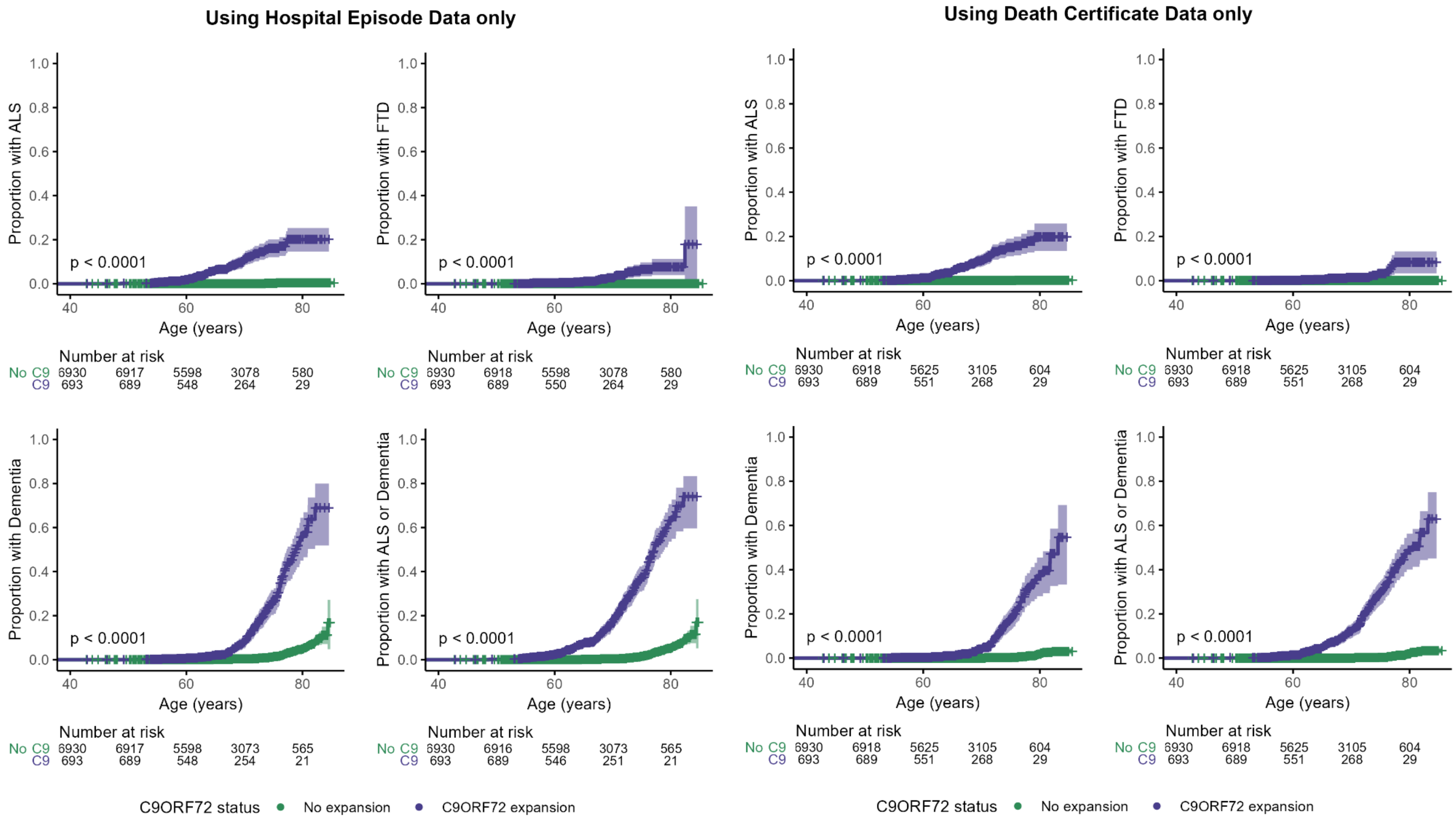

Supplementary Figure 5 – Age-related cumulative incidences of ALS and dementias in carriers of the *C9ORF72* HRE compared to matched controls by data source

Cumulative incidences of ALS and dementia by age in individuals carrying the *C9ORF72* HRE ("C9", n =693) compared to age, sex, Townsend deprivation index and ethnicity matched controls ("No C9", n= 6930), using only hospital episode data derived diagnoses (left) or only death certificate diagnoses (right).

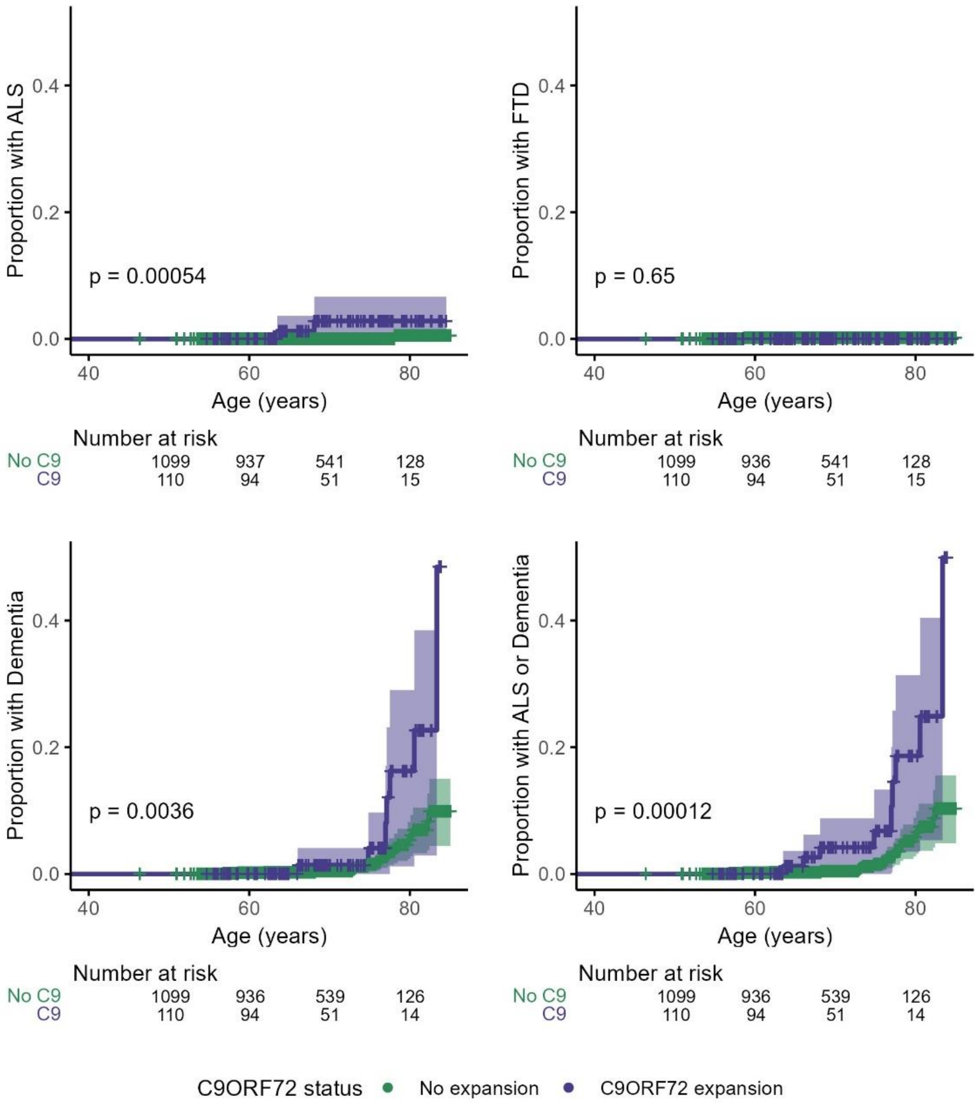

Supplementary Figure 6 - Age-related cumulative incidences of ALS and dementias in carriers of 30-100 repeats in *C9ORF72* compared to matched controls, using Kaplan-Meier models

Cumulative incidences of ALS, FTD, dementia and a combined outcome of ALS and dementia by age in individuals carrying 30-100 repeats in *C9ORF72* (purple, n =110) compared to age, sex, Townsend deprivation index and ethnicity matched controls (green, n= 1100), using Kaplan Meier models. Shaded bands indicate 95% confidence intervals.

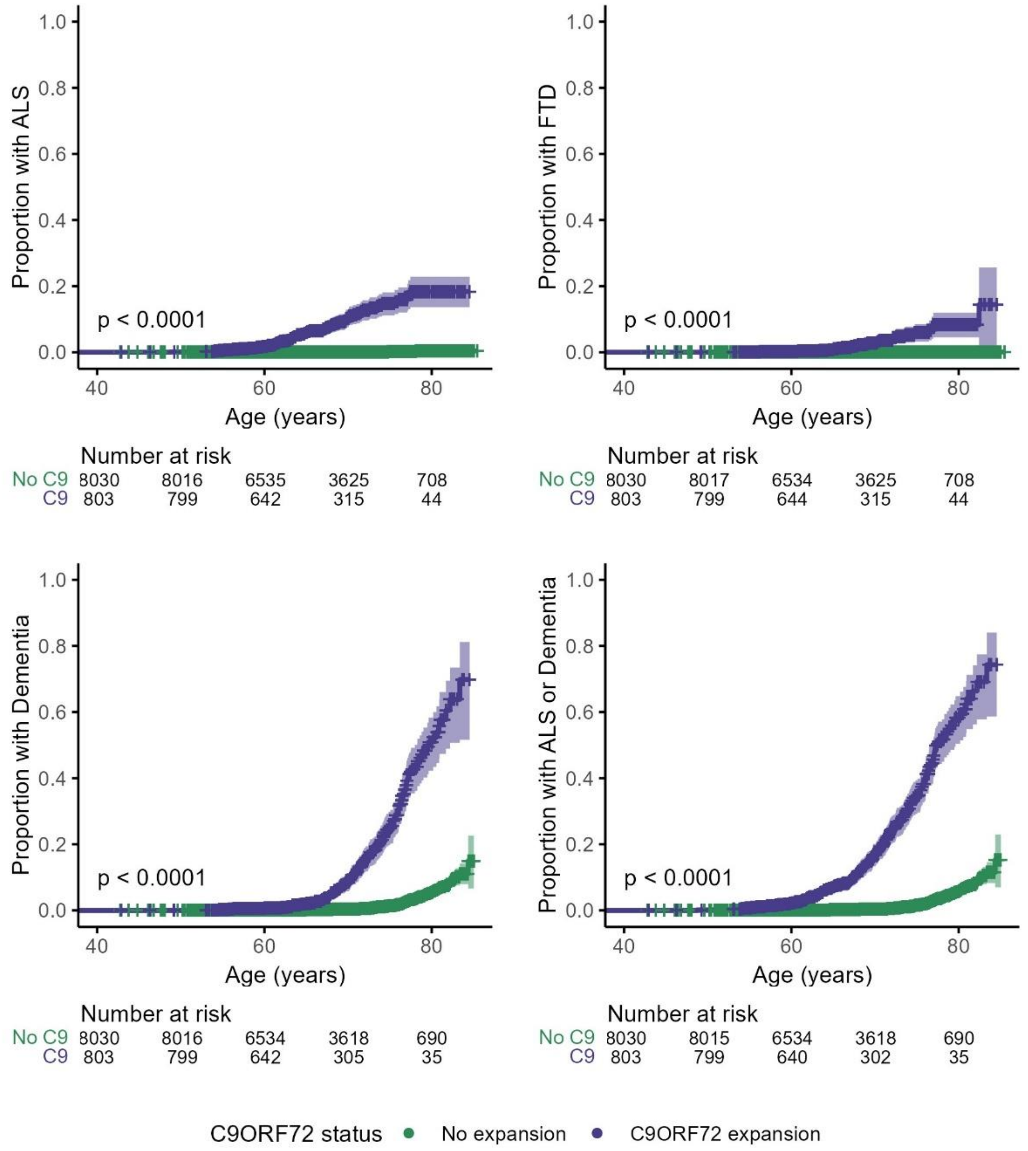

Supplementary Figure 7 - Age-related cumulative incidences of ALS and dementias in carriers of >30 repeats in *C9ORF72* compared to matched controls, using Kaplan-Meier models

Cumulative incidences of ALS, FTD, dementia and a combined outcome of ALS and dementia by age in individuals carrying >30 repeats in *C9ORF72* (purple, n =803) compared to age, sex, Townsend deprivation index and ethnicity matched controls (green, n= 8030), using Kaplan Meier models. Shaded bands indicate 95% confidence intervals.

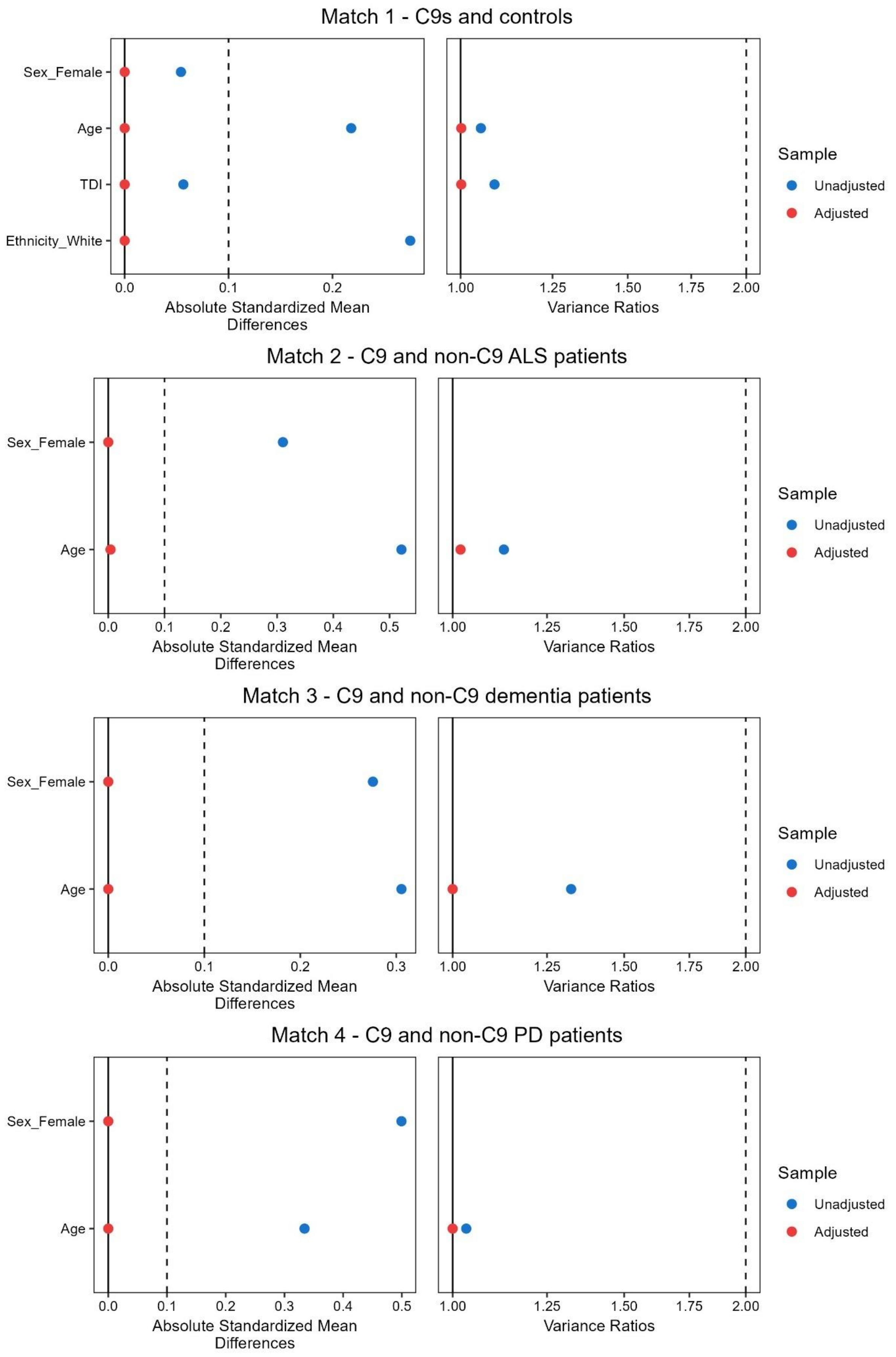

Supplementary Figure 8 – Love plots of covariate balance before and after matching

Love plots of absolute standardised mean differences and variance ratios between *C9ORF72* HRE carriers and controls before (blue) and after (red) matching

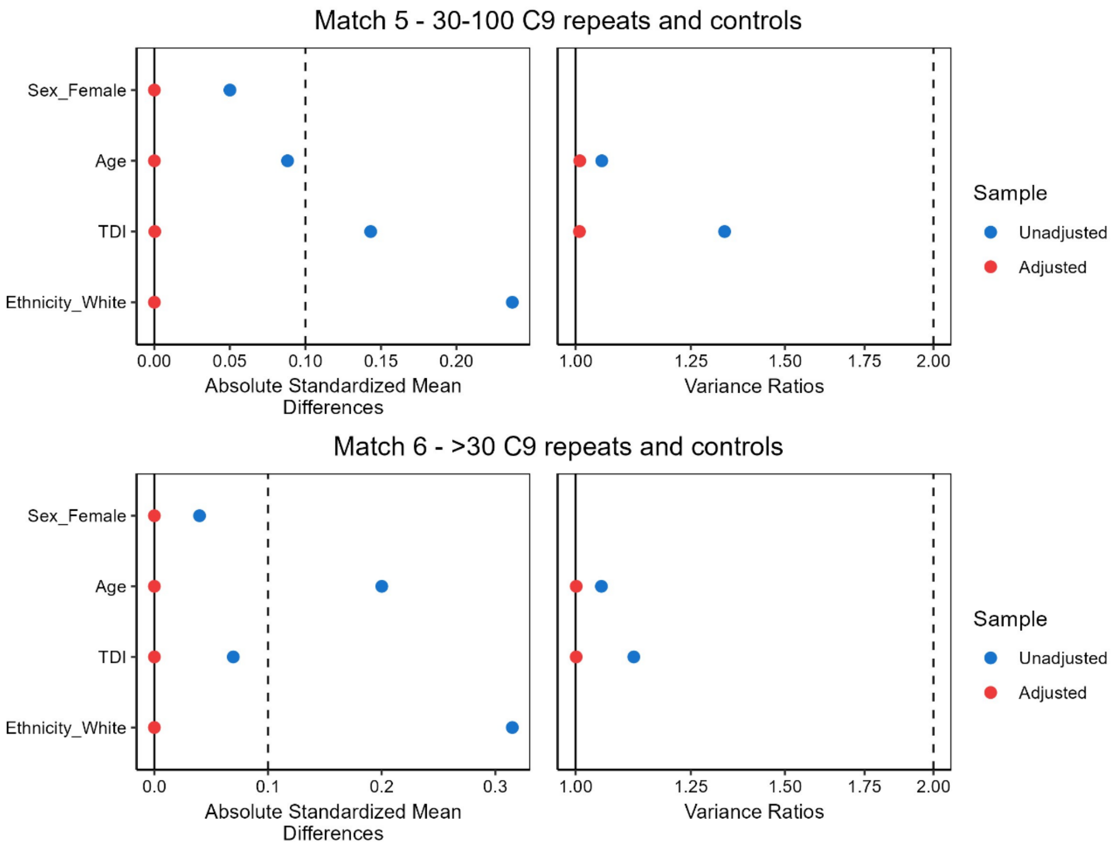

Supplementary Figure 8 – Love plots of covariate balance before and after matching (cont)

Love plots of absolute standardised mean differences and variance ratios between *C9ORF72* HRE carriers and controls before (blue) and after (red) matching

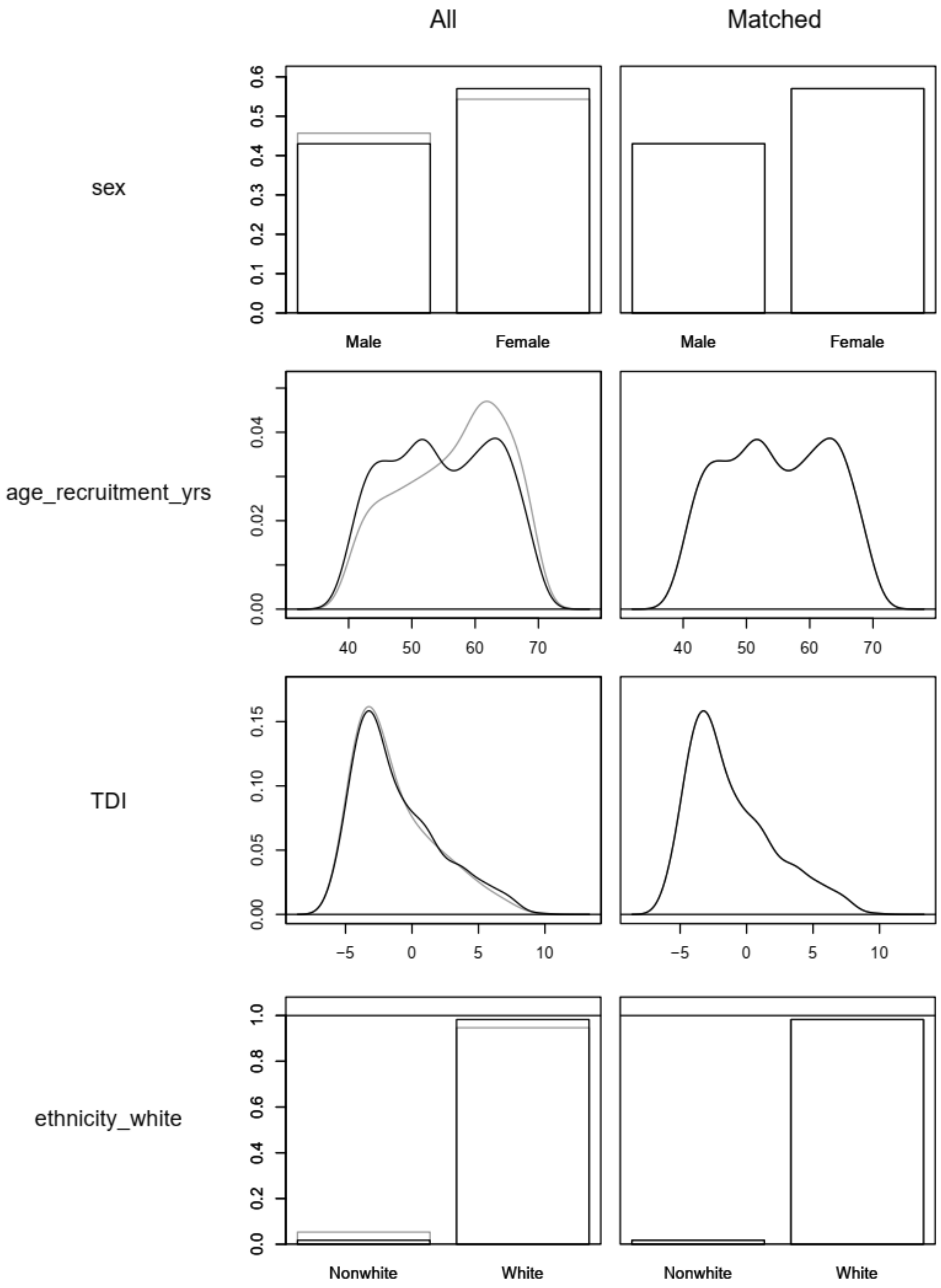

Supplementary Figure 9 – Density plots of covariate balance before and after matching in *C9ORF72* HRE carriers and controls for cumulative incidence calculations (Match 1)

Density plots of matching variables before (left) and after (right) matching in *C9ORF72* HRE carriers (black) and controls (grey). Age_recruitment_yrs = Age at recruitment in years, TDI = Townsend deprivation index, ethnicity_white = ethnicity categories as white and non-white.

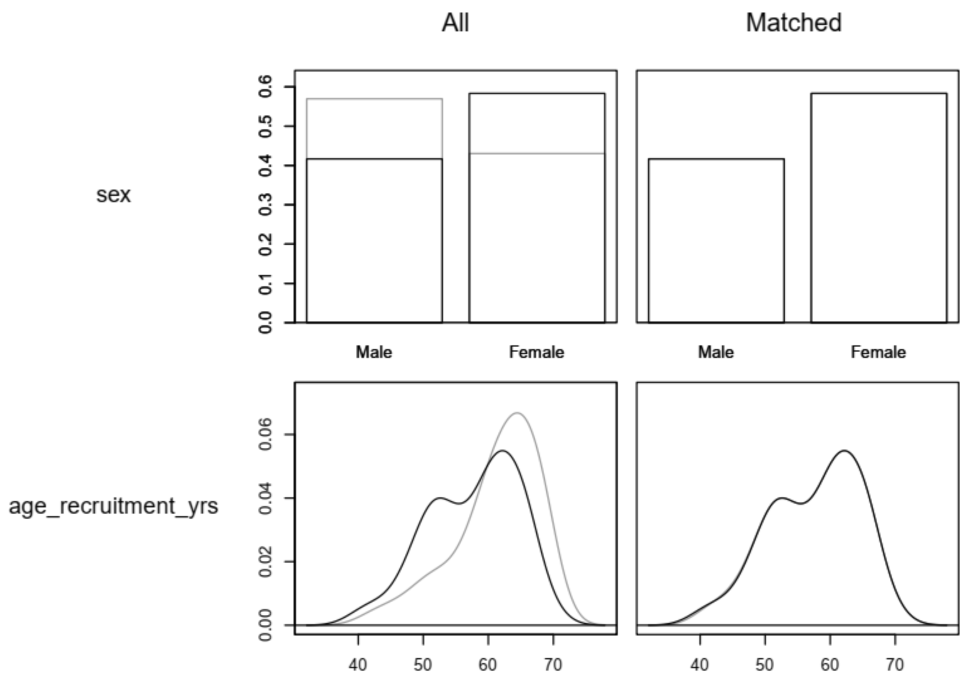

Supplementary Figure 10 – Density plots of covariate balance before and after matching in *C9ORF72* HRE and non-*C9ORF72* ALS patients for age of onset comparison (Match 2)
Density plots of matching variables before (left) and after (right) matching in *C9ORF72* HRE carriers (black) and controls (grey). Age_recruitment_yrs = Age at recruitment in years

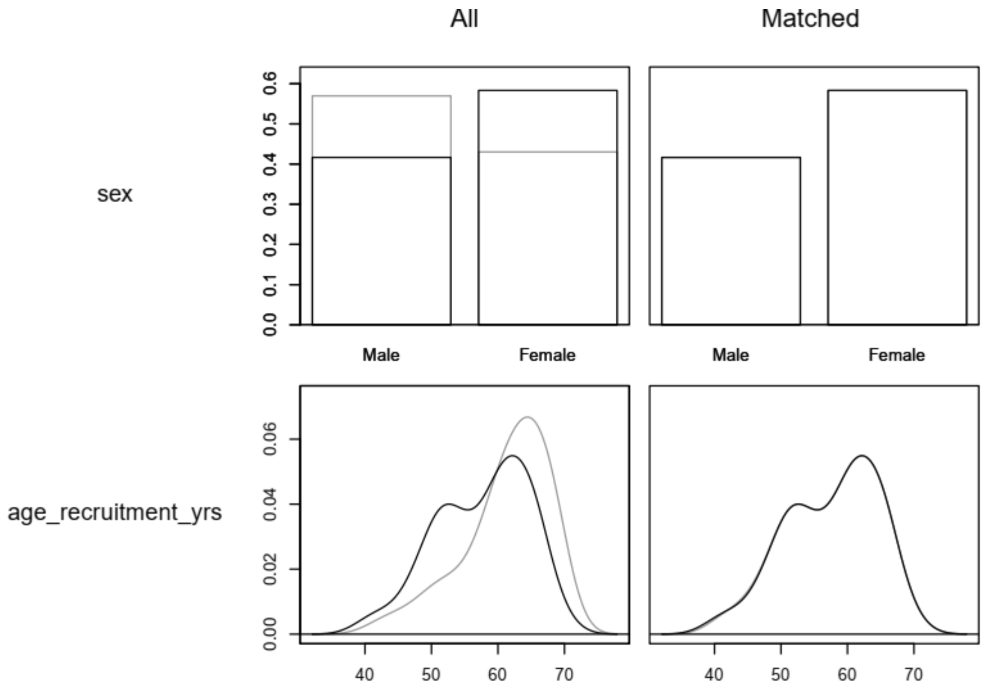

Supplementary Figure 11 – Density plots of covariate balance before and after matching in *C9ORF72* HRE and non-*C9ORF72* dementia patients for age of onset comparison (Match 3)
Density plots of matching variables before (left) and after (right) matching in *C9ORF72* HRE carriers (black) and controls (grey). Age_recruitment_yrs = Age at recruitment in years

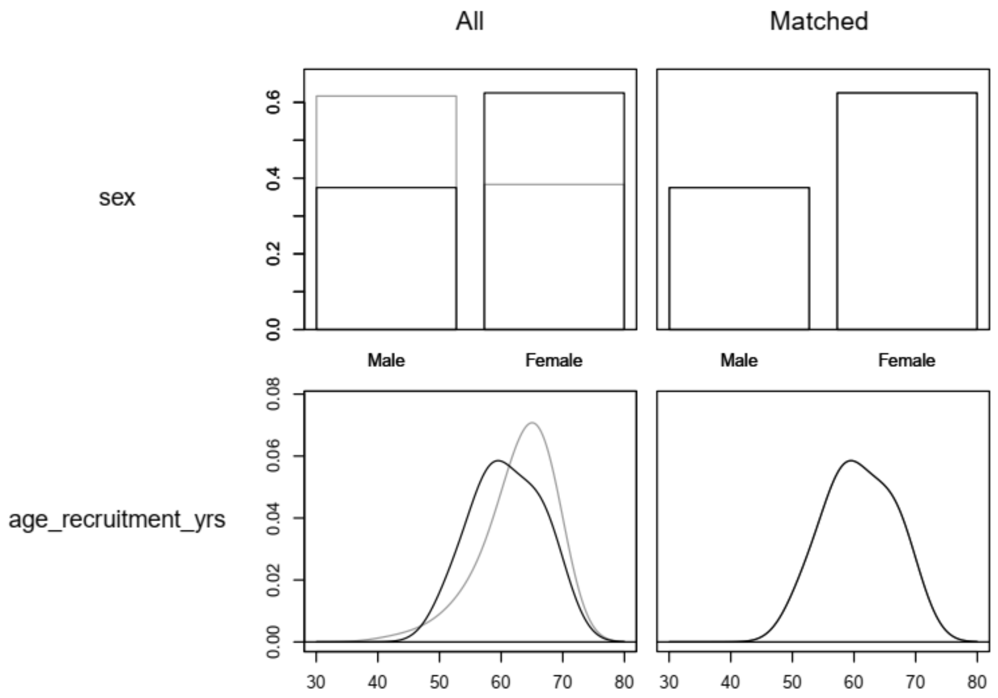

Supplementary Figure 12 – Density plots of covariate balance before and after matching in *C9ORF72* HRE and non-*C9ORF72* parkinsonism patients for age of onset comparison (Match 4)

Density plots of matching variables before (left) and after (right) matching in *C9ORF72* HRE carriers (black) and controls (grey). Age_recruitment_yrs = Age at recruitment in years

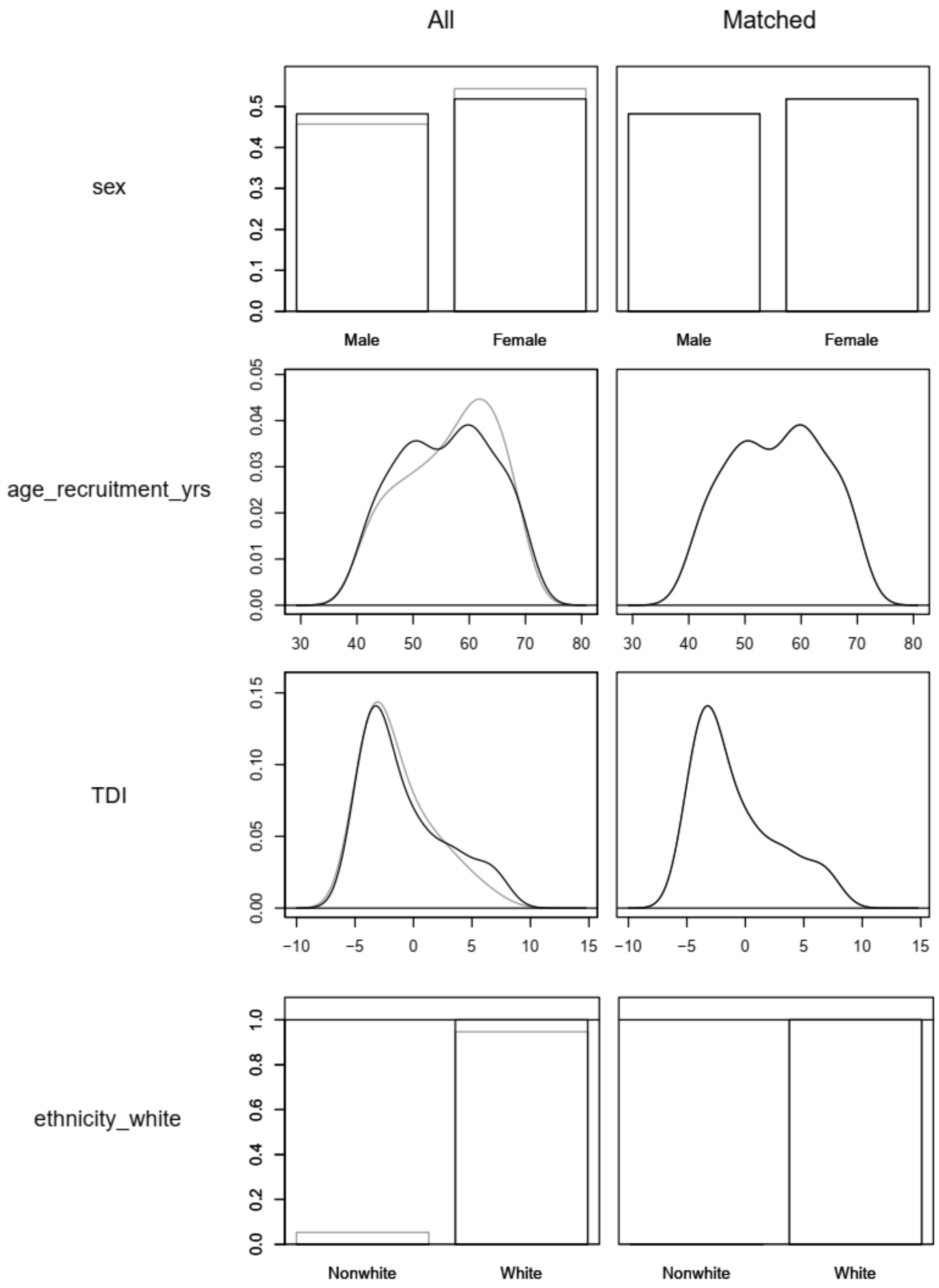

Supplementary Figure 13 – Density plots of covariate balance before and after matching in those with 30-100 repeats in C9ORF72 and controls for cumulative incidence calculations (Match 5)

Density plots of matching variables before (left) and after (right) matching in participants with 30-100 repeats in *C9ORF72* (black) and controls (grey). Age_recruitment_yrs = Age at recruitment in years, TDI = Townsend deprivation index, ethnicity_white = ethnicity categories as white and non-white.

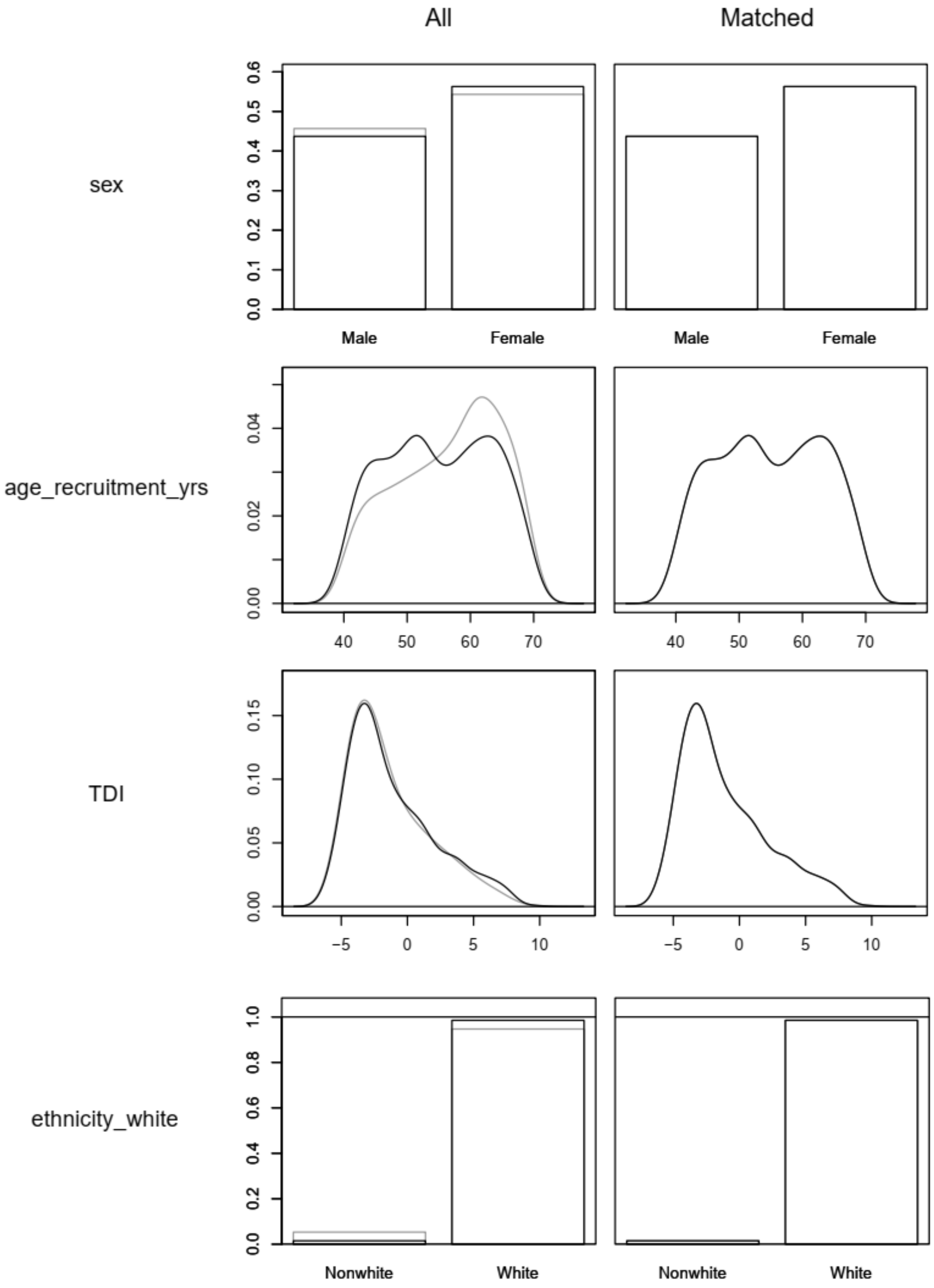

Supplementary Figure 14 – Density plots of covariate balance before and after matching in those with >30 repeats in C9ORF72 and controls for cumulative incidence calculations (Match 6)

Density plots of matching variables before (left) and after (right) matching in participants with >30 repeats in *C9ORF72* (black) and controls (grey). Age_recruitment_yrs = Age at recruitment in years, TDI = Townsend deprivation index, ethnicity_white = ethnicity categories as white and non-white.

References

1. Li S, Carss KJ, Halldorsson BV, Cortes A, Consortium UBWGS. Whole-genome sequencing of half-a-million UK Biobank participants. Published online December 8, 2023:2023.12.06.23299426. doi:10.1101/2023.12.06.23299426

2. Dolzhenko E, Vugt JJFA van, Shaw RJ, et al. Detection of long repeat expansions from PCR-free whole-genome sequence data. *Genome Res*. 2017;27(11):1895-1903. doi:10.1101/gr.225672.117

3. ExpansionHunter/variant_catalog/grch38 at master · Illumina/ExpansionHunter · GitHub. Accessed June 22, 2024. https://github.com/Illumina/ExpansionHunter/tree/master/variant_catalog/grch38

4. Weidburd B, VanNoy G, Watts N. The Addition of Short Tandem Repeat Calls to gnomAD (v3.1.3). January 21, 2022. https://gnomad.broadinstitute.org/news/2022-01-the-addition-of-short-tandem-repeat-calls-to-gnomad/

5. Dolzhenko E, Weisburd B, Ibañez K, et al. REViewer: haplotype-resolved visualization of read alignments in and around tandem repeats. *Genome Medicine*. 2022;14(1):84. doi:10.1186/s13073-022-01085-z

6. Pottier C, Ren Y, Perkerson RB, et al. Genome-wide analyses as part of the international FTLD-TDP whole-genome sequencing consortium reveals novel disease risk factors and increases support for immune dysfunction in FTLD. *Acta Neuropathol*. 2019;137(6):879-899. doi:10.1007/s00401-019-01962-9

7. Machiela MJ, Chanock SJ. LDlink: a web-based application for exploring population-specific haplotype structure and linking correlated alleles of possible functional variants. *Bioinformatics*. 2015;31(21):3555-3557. doi:10.1093/bioinformatics/btv402

STROBE Statement—checklist of items that should be included in reports of observational studies

	Item No.	Recommendation	Page No.	Relevant text from manuscript
**Title and abstract**	1	(*a*) Indicate the study's design with a commonly used term in the title or the abstract	1	
		(*b*) Provide in the abstract an informative and balanced summary of what was done and what was found	1	
**Introduction**				
Background/rationale	2	Explain the scientific background and rationale for the investigation being reported	2-3	
Objectives	3	State specific objectives, including any prespecified hypotheses	3	
**Methods**				
Study design	4	Present key elements of study design early in the paper	3-4	
Setting	5	Describe the setting, locations, and relevant dates, including periods of recruitment, exposure, follow-up, and data collection	3-4	
Participants	6	(*a*) *Cohort study*—Give the eligibility criteria, and the sources and methods of selection of participants. Describe methods of follow-up<br>*Case-control study*—Give the eligibility criteria, and the sources and methods of case ascertainment and control selection. Give the rationale for the choice of cases and controls<br>*Cross-sectional study*—Give the eligibility criteria, and the sources and methods of selection of participants	3-4	
		(*b*) *Cohort study*—For matched studies, give matching criteria and number of exposed and unexposed<br>*Case-control study*—For matched studies, give matching criteria and the number of controls per case	Suppl 4-5	
Variables	7	Clearly define all outcomes, exposures, predictors, potential confounders, and effect modifiers. Give diagnostic criteria, if applicable	Suppl 3-5	

Data sources/ measurement	8*	For each variable of interest, give sources of data and details of methods of assessment (measurement). Describe comparability of assessment methods if there is more than one group	Supplementary Table 1
Bias	9	Describe any efforts to address potential sources of bias	3-4
Study size	10	Explain how the study size was arrived at	NA
Quantitative variables	11	Explain how quantitative variables were handled in the analyses. If applicable, describe which groupings were chosen and why	4
Statistical methods	12	(*a*) Describe all statistical methods, including those used to control for confounding	4, Suppl 3-5
		(*b*) Describe any methods used to examine subgroups and interactions	4
		(*c*) Explain how missing data were addressed	4
		(*d*) *Cohort study*—If applicable, explain how loss to follow-up was addressed<br>*Case-control study*—If applicable, explain how matching of cases and controls was addressed<br>*Cross-sectional study*—If applicable, describe analytical methods taking account of sampling strategy	4
		(*e*) Describe any sensitivity analyses	3-4, Suppl 3-5
Results			
Participants	13*	(a) Report numbers of individuals at each stage of study—eg numbers potentially eligible, examined for eligibility, confirmed eligible, included in the study, completing follow-up, and analysed	5-7
		(b) Give reasons for non-participation at each stage	5-7
		(c) Consider use of a flow diagram	NA
Descriptive data	14*	(a) Give characteristics of study participants (eg demographic, clinical, social) and information on exposures and potential confounders	Supplementary Table2-3
		(b) Indicate number of participants with missing data for each variable of interest	Supplementary Table2-3

		(c) *Cohort study*—Summarise follow-up time (eg, average and total amount)	Supplementary Table2-3
Outcome data	15*	*Cohort study*—Report numbers of outcome events or summary measures over time	5, Fig1
		Case-control study—Report numbers in each exposure category, or summary measures of exposure	
		Cross-sectional study—Report numbers of outcome events or summary measures	
Main results	16	(*a*) Give unadjusted estimates and, if applicable, confounder-adjusted estimates and their precision (eg, 95% confidence interval). Make clear which confounders were adjusted for and why they were included	5-7
		(*b*) Report category boundaries when continuous variables were categorized	NA
		(*c*) If relevant, consider translating estimates of relative risk into absolute risk for a meaningful time period	NA
Other analyses	17	Report other analyses done—eg analyses of subgroups and interactions, and sensitivity analyses	5-7
**Discussion**			
Key results	18	Summarise key results with reference to study objectives	7
Limitations	19	Discuss limitations of the study, taking into account sources of potential bias or imprecision. Discuss both direction and magnitude of any potential bias	7-9
Interpretation	20	Give a cautious overall interpretation of results considering objectives, limitations, multiplicity of analyses, results from similar studies, and other relevant evidence	8-9
Generalisability	21	Discuss the generalisability (external validity) of the study results	8-9
**Other information**			
Funding	22	Give the source of funding and the role of the funders for the present study and, if applicable, for the original study on which the present article is based	10

*Give information separately for cases and controls in case-control studies and, if applicable, for exposed and unexposed groups in cohort and cross-sectional studies.
